# A decade of submersible observations revealed temporal trends in elasmobranchs in a remote island of the Eastern Tropical Pacific Ocean

**DOI:** 10.1038/s41598-024-64157-7

**Published:** 2024-06-14

**Authors:** Mario Espinoza, Fabio Quesada-Perez, Sergio Madrigal-Mora, Beatriz Naranjo-Elizondo, Tayler M. Clarke, Jorge Cortés

**Affiliations:** 1 Centro de Investigación en Ciencias del Mar y Limnología, Universidad de Costa Rica, San Pedro, San José 11501-2060 Costa Rica; 2 Escuela de Biología, Universidad de Costa Rica, San Pedro, San José 11501-2060 Costa Rica; 3 MigraMar, Bodega Bay, CA 94923 USA; 4 Shark Lab, California State University, Long Beach, CA 90840 USA; 5 Centro de Investigación en Estructuras Microscópicas (CIEMic), Universidad de Costa Rica, San Pedro, San José 11501-2060 Costa Rica; 6 Pelagos Okeanos, Moravia, San José 11401 Costa Rica; 7 Changing Ocean Research Unit, Institute for the Oceans and Fisheries, University of British Columbia, AERL, 2202 Main Mall, Vancouver, BC V6T 1Z4 Canada

**Keywords:** No-take marine protected areas, Isla del Coco National Park, Deepwater biodiversity, Elasmobranchs, Ocean warming, Biodiversity, Climate-change ecology, Community ecology, Conservation biology, Population dynamics, Ichthyology, Biodiversity

## Abstract

No-take marine protected areas (MPAs) can mitigate the effects of overfishing, climate change and habitat degradation, which are leading causes of an unprecedented global biodiversity crisis. However, assessing the effectiveness of MPAs, especially in remote oceanic islands, can be logistically challenging and often restricted to relatively shallow and accessible environments. Here, we used a long-term dataset (2010–2019) collected by the *DeepSee* submersible of the Undersea Hunter Group that operates in Isla del Coco National Park, Costa Rica, to (1) determine the frequency of occurrence of elasmobranch species at two depth intervals (50–100 m; 300–400 m), and (2) investigate temporal trends in the occurrence of common elasmobranch species between 2010 and 2019, as well as potential drivers of the observed changes. Overall, we observed 17 elasmobranch species, 15 of which were recorded on shallow dives (50–100 m) and 11 on deep dives (300–400 m). We found a decreasing trend in the probability of occurrence of *Carcharhinus falciformis* over time (2010–2019), while other species (e.g. *Taeniurops meyeni*, *Sphyrna lewini*, *Carcharhinus galapagensis*, *Triaenodon obesus*, and *Galeocerdo cuvier*) showed an increasing trend. Our study suggests that some species like *S. lewini* may be shifting their distributions towards deeper waters in response to ocean warming but may also be sensitive to low oxygen levels at greater depths. These findings highlight the need for regional 3D environmental information and long-term deepwater surveys to understand the extent of shark and ray population declines in the ETP and other regions, as most fishery-independent surveys from data-poor countries have been limited to relatively shallow waters.

## Introduction

Anthropogenic impacts are responsible for the unprecedented global biodiversity crisis affecting both terrestrial and aquatic environments^1–3^. Overfishing is the main cause of the increased risk of extinction that threatens a third of all sharks and rays, while other stressors such as climate change and habitat degradation are believed to have a lesser impact^4,5^. Large declines in shark and ray populations are expected to have negative impacts on the structure and function of aquatic food webs, as high trophic level predators play important ecological roles that help maintain ecosystem health^6,7^. This creates an urgent need to mitigate the impacts of fishing and increase climate resilience, which is being partly addressed through the establishment of extensive Marine Protected Areas (MPAs), such as those in the Eastern Tropical Pacific Ocean Marine Corridor (CMAR)^8^. Evaluating the results of implementing these MPA’s is vital to ensure they are effectively protecting vulnerable species (e.g. sharks, rays, sea turtles) and preserving ecosystem health. In the face of data scarcity and financial challenges, it is crucial to move beyond fisheries data to explore innovative avenues of monitoring and improving conservation and management strategies^9,10^.

Citizen science programs are becoming a popular and cost-effective tool for scientists and managers working in marine environments, offering a path to improve our current knowledge of marine biodiversity and supporting marine conservation efforts^11^. Such programs enable citizens to participate in scientific projects with different scopes and scales, thus allowing collection of large datasets that can help answer a wide range of questions^12,13^. However, it is important to ensure that data collection by untrained observers is robust and reliable^13^. For example, in underwater visual surveys, species identification and counts can vary considerably among divers with different degrees of experience and field training, yet the quality and quantity of data collected can be improved with the use of photographic/video records^14^. Moreover, the identification of large iconic elasmobranch species (e.g., whale sharks and giant manta rays) or species with distinct markings and shapes (e.g., tiger and hammerhead sharks, eagle rays) is typically more reliable because it is easier to learn by divers, particularly those that frequently visit the same dive sites^15,16^. Consequently, as overfishing and climate change continues to impact marine species and ecosystems across the globe, well-designed citizen science efforts can complement scientific surveys of marine species in data-poor regions.

Long-term records of elasmobranch species occurrence and abundance collected by citizen scientists can help assess population-level changes in response to common threats such as overfishing and ocean warming, which ultimately can guide management and conservation actions^16^. For example, dive masters from Undersea Hunter Group, a tour-operator company based in Costa Rica, recorded sightings of key marine megafauna (sharks, rays, sea turtles, dolphins, tuna, etc.) at Isla del Coco National Park (hereafter referred to as Isla del Coco) between 1991 and the present, which represents one of the most complete long-term datasets available in a remote and relatively pristine oceanic island from the Eastern Tropical Pacific Ocean. This dataset has allowed scientists to investigate distribution patterns^17,18^, species-habitat and species-environmental relationships^19,20^, and population trends^19,21^ of elasmobranchs in the shallow waters of Isla del Coco. These studies revealed significant declines in the relative abundance of multiple elasmobranch species that were mainly explained by overfishing, El Niño events and ocean warming^19–21^.

Warming waters around Isla del Coco may be forcing sharks and rays into cooler, deeper waters^19^, but the magnitude of this shift in species’ depth distributions may be constrained by the shallow upper boundary of the oxygen minimum zone^22^. Consistent video records of elasmobranch sightings between 50 and 400 m collected by the Undersea Hunter *DeepSee* submersible provide a unique opportunity to study population trends in these relatively deeper habitats^23^ and analyze the effects of ocean warming and the expansion of the oxygen minimum zone on their depth distribution. *DeepSee* has also enabled the study of complex behavioral interactions in deep water predatory fish^24^. Analyzing elasmobranch trends in deeper waters can provide valuable insights into species diversity and distribution in less accessible environments and complete the picture of how climate change may be affecting them. This study used a ten-year sighting database (2010–2019) from the Undersea Hunter *DeepSee* submersible operating at Isla del Coco to (1) determine the frequency of occurrence of elasmobranch species at two depth intervals (50–100 m; 300–400 m), and (2) investigate temporal trends in the occurrence of common elasmobranch species between 2010 and 2019, as well as potential drivers of the observed changes.

## Results

### Frequency of elasmobranch occurrence

**Figure 1:**
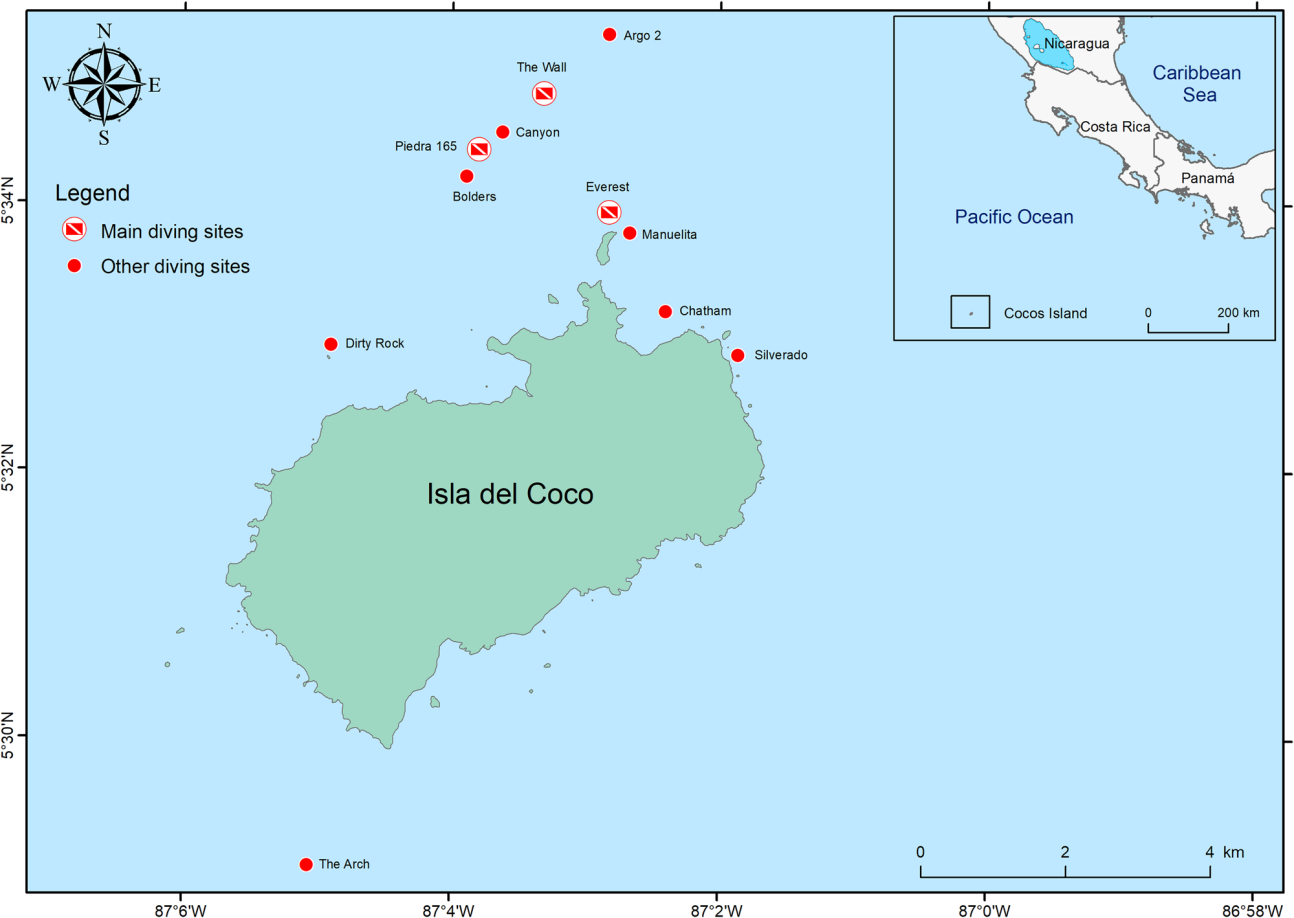
Map of the *DeepSee* submersible dive sites in Isla del Coco National Park, Costa Rica. From 2009 to 2019, the *DeepSee* regularly conducted dives at Everest, Piedra 165 and The Wall, but during this period it also surveyed additional sites. Dive sites were plotted using base layers from the Costa Rican Geographic Information System (GIS) Atlas open-access project (http://hdl.handle.net/2238/6749) in ArcMap 10.4 (ESRI, Redlands, California).

**Figure 2:**
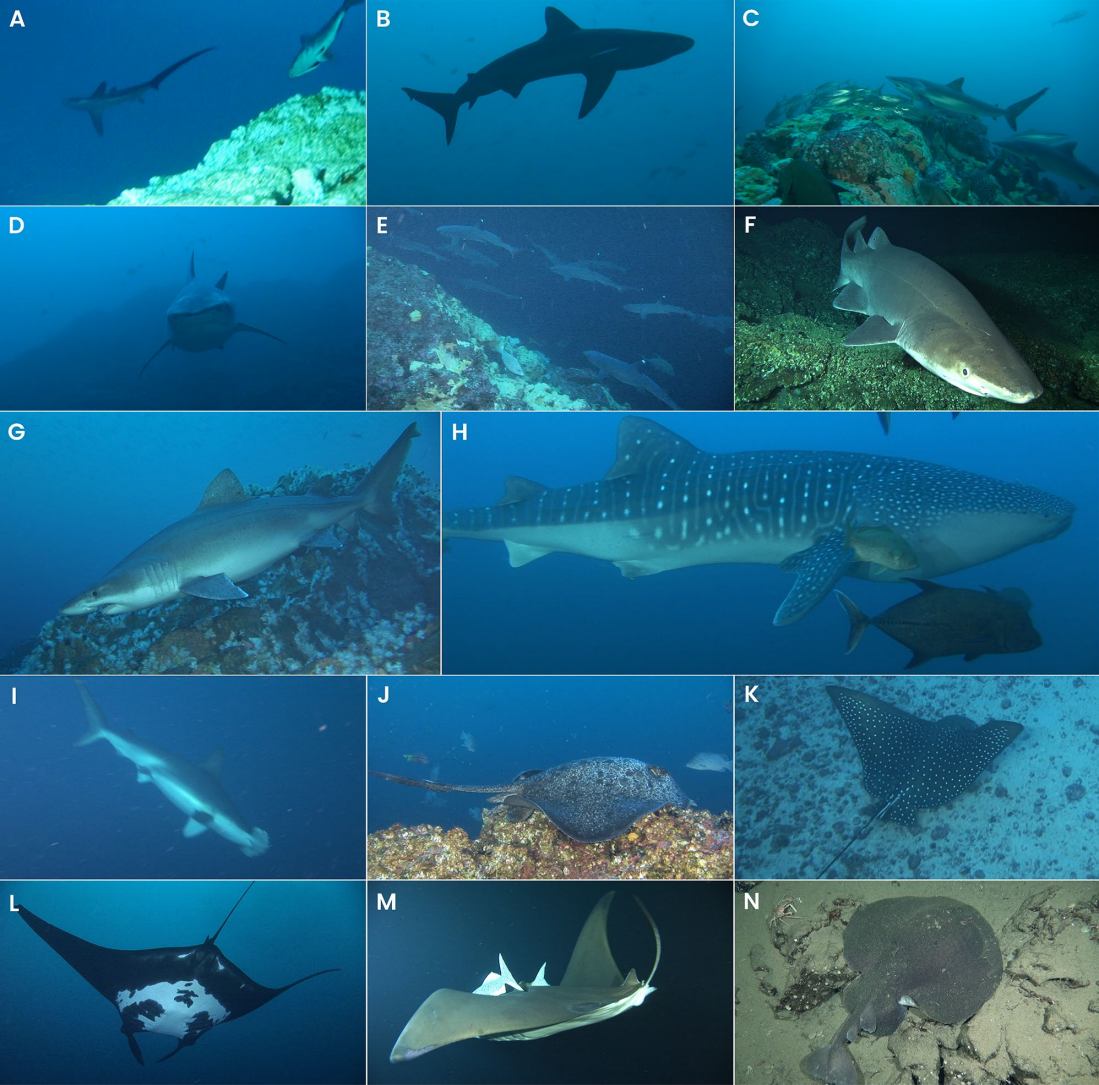
Elasmobranch species sighted during the *DeepSee* submersible dives in Isla del Coco National Park between 2010 and 2019. Species: (**A**) *Alopias* sp., (**B**) *Carcharhinus falciformis*, (**C**) *C. galapagensis*, (**D**) *Galeocerdo cuvier*, (**E**) *Triaenodon obesus*, (**F**) *Echinorhinus cookei*, (**G**) *Odontaspis ferox*, (**H**) *Rhincodon typus*, (**I**) *Sphyrna lewini*, (**J**) *Taeniurops meyeni*, (**K**) *Aetobatus laticeps*, (**L**) *Mobula birostris*, (**M**) *M. tarapacana*, (**N**) *Tetronarce tremens*. All photos were taken during submersible dives and are property of the Undersea Hunter Group.

Between 2010 and 2019, a total of 1573 submersible dives were conducted on Isla del Coco (Fig. 1), of which 428 dives were at depths of 50–100 m and 1145 dives were at depths of 300–400 m. During these dives, we recorded a total of 17 elasmobranch species from nine families (Table 1, Fig. 2). Elasmobranchs were observed on 82% of the dives, with the number of species recorded per dive ranging from 0 to 7 (mean ± SD: 2 ± 1 species). The Giant oceanic manta ray (*Mobula birostris*) and the Chilean devil ray (*M. tarapacana*) occurred in approximately 57% of the dives and were the most common group recorded. The Scalloped hammerhead (*Sphyrna lewini*), Silky (*Carcharhinus falciformis*), and Galapagos (*C*. *galapagensis*) sharks were sighted in 40%, 19% and 12% of the dives, respectively. The remaining elasmobranch species were seen on less than 5% of the dives.

**Table 1:** Elasmobranch species recorded by the *DeepSee* submersible at Isla del Coco, Eastern Tropical Pacific Ocean.

Family	Species	Common name	IUCN red list	CITES appendix
Alopiidae	*Alopias* spp.	Thresher shark	VU/EN	II (2017)
Carcharhinidae	*Carcharhinus albimarginatus*	Silvertip shark	VU	–
	*Carcharhinus galapagensis*	Galapagos shark	LC	–
	*Carcharhinus falciformis*	Silky shark	VU	II (2017)
	*Carcharhinus limbatus*	Blacktip shark	VU	–
	*Galeocerdo cuvier*	Tiger shark	NT	–
	*Triaenodon obesus*	Whitetip reef shark	VU	–
Echinorhinidae	*Echinorhinus cookei*	Prickly shark	DD	–
Odontaspididae	*Odontaspis ferox*	Smalltooth sand tiger	VU	–
Rhincodontidae	*Rhincodon typus*	Whale shark	EN	II (2003)
Sphyrnidae	*Sphyrna lewini*	Scalloped hammerhead	CR	II (2014)
Dasyatidae	*Taeniurops meyeni*	Blotched fantail ray	VU	–
Myliobatidae	*Aetobatus laticeps*	Pacific eagle ray	VU	–
	*Mobula birostris*	Giant Oceanic manta ray	EN	II (2014)
	*Mobula tarapacana*	Chilean devil ray	EN	II (2016)
	*Rhinoptera steindachneri*	Golden cownose ray	NT	–
Torpedinidae	*Tetronarce tremens*	Chilean torpedo ray	LC	–

IUCN (The International Union for Conservation of Nature) Red List Status: DD—Data Deficient; LC—Least Concern; NT—Near Threatened; VU—Vulnerable; EN—Endangered; CR—Critically Endangered. CITES (The Convention on International Trade in Endangered Species of Wild Fauna and Flora) Appendix II lists species that are not necessarily threatened with extinction but that may become so unless trade is closely controlled.

**Figure 3:**
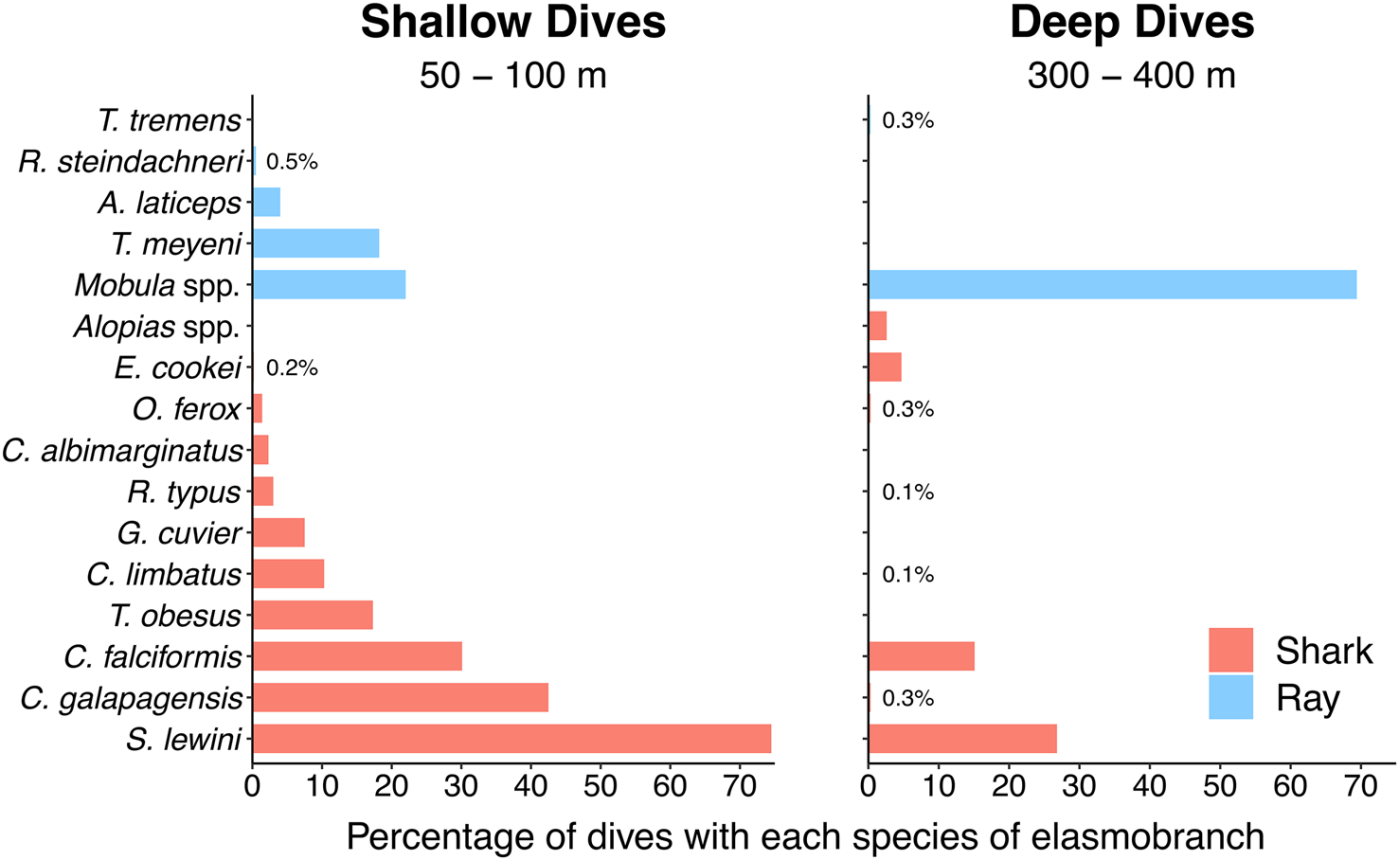
Percentage of *DeepSee* submersible dives in which each elasmobranch species was sighted at Isla del Coco, Eastern Tropical Pacific Ocean, by maximum depth interval. Species that were sighted in less than 0.6% of the dives are shown as text.

More elasmobranch species were recorded on shallow dives (14 species) than on deep dives (10 species) (Fig. 3). The most common species on shallow dives was *S. lewini*, recorded on 75% of the dives, followed by *C. galapagensis* (43% of the dives) and *C. falciformis* (30% of the dives) (Fig. 3). *Mobula* spp. were recorded on 69% of the deep dives. Other species such as Tiger (*Galeocerdo cuvier*) and Whitetip Reef (*Triaenodon obesus*) sharks were recorded exclusively on shallow dives, while Thresher sharks (*Alopias* spp.) and the Chilean Torpedo rays (*Tetronarce tremens*) were only recorded on deep dives (Fig. 3).

### Temporal trends of elasmobranchs

**Figure 4:**
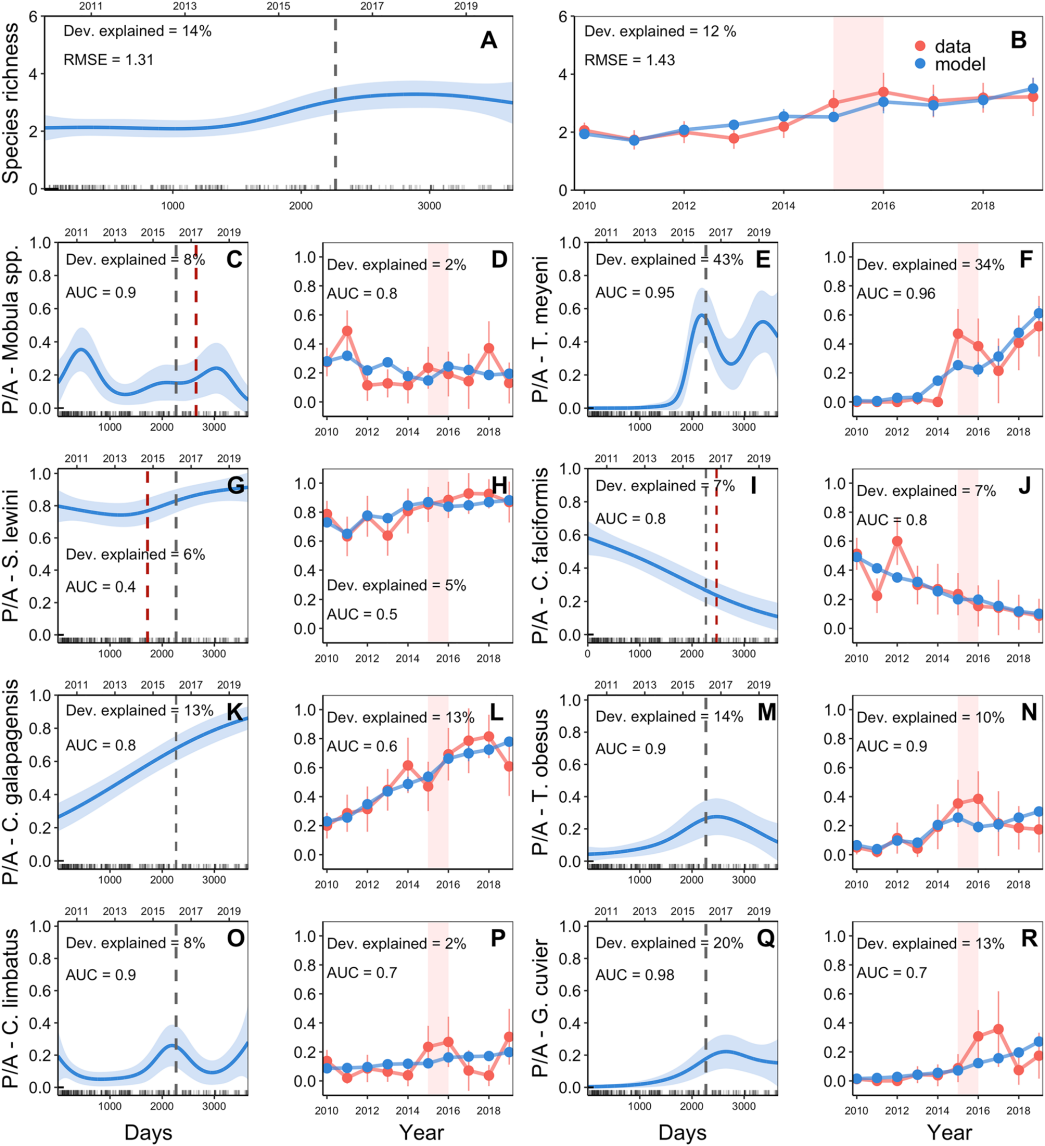
GAM estimates of daily species richness and probability of occurrence of elasmobranch species for depths between 50 and 100 m (**A**, **C**, **E**, **G**, **I**, **K**, **M**, **O** & **Q**). The x-axis indicates the number of days relative to the start of the study (January 1, 2010). Estimated smooth functions (solid lines) with 95% confidence intervals (shaded areas) are shown for each numerical explanatory variable. The dates when a surveillance radar was installed on Cocos Island (dashed grey line) and when the species was listed in CITES Appendix II (dashed red line) are shown as reference points. Observed data and GLM model estimates of mean annual species richness and probability of occurrence of elasmobranch species from 2010 to 2019 for depths between 50 and 100 m (**B**, **D**, **F**, **H**, **J**, **L**, **N**, **P** & **R**). Bars represent the 95% confidence intervals. Red rectangles indicate the El Niño event of 2015–2016.

Generalized Additive Models (GAMs) revealed significant effects of temporal and environmental drivers on elasmobranch species richness in dives from 50 to 100 m. The model with the highest goodness-of-fit included day, SST anomalies, and time period (morning/afternoon) (Table 2). The predicted number of species sighted exhibited a significant increase from 2013 to 2017, followed by a slight decrease until 2019 (Fig. 4A). Furthermore, the number of elasmobranch species recorded increased under warmer SST anomalies and during afternoon hours (see Supplement S1). The Generalized Linear Model (GLM) that best explained species richness in shallow dives included year, SST anomalies and time period (Table 3). Based on this model, the estimated number of elasmobranchs recorded in shallow dives increased from approximately two species in 2010 to four species in 2019 (Fig. 4B).

**Table 2:** Summary of generalized additive models for species richness and presence-absence data of elasmobranch species sighted during shallow dives (50–100 m) at Isla del Coco, Eastern Pacific Ocean.

Error distribution	Response	Predictors	% deviance explained	RMSE	AUC
Poisson	Richness	s(days)* + SST anomalies* + time period* + offset (log (dive time))	14	1.31	–
Binomial	P_*Mobula* spp._	s(days)* + time period* + offset (log (dive time))	8	–	0.9
	P_*T. meyeni*_	s(days)* + offset (log (dive time))	43		0.95
	P_*S. lewini*_	s(days)* + SST anomalies* + offset (log (dive time))	6	–	0.4
	P_*C. falciformis*_	s(days)* + offset (log (dive time))	7	–	0.8
	P_*C. galapagensis*_	s(days)* + season* + offset (log (dive time))	13	–	0.8
	P_*T. obesus*_	s(days)* + SST anomalies* + offset (log (dive time))	13	–	0.9
	P_*C. limbatus*_	s(days)* + offset (log (dive time))	8	–	0.9
	P_*G. cuvier*_	s(days)* + offset (log (dive time))	20	–	0.98

Asterisk indicates statistical significance, p < 0.05.

**Table 3:** Summary of binomial Generalized Linear Models for species richness and presence-absence data of elasmobranch species sighted during shallow dives (50–100 m) at Isla del Coco, Eastern Tropical Pacific Ocean.

Error distribution	Response	Predictors	% deviance explained	RMSE	AUC
Poisson	Richness	year* + SST anomalies* + time period * + offset (log (dive time))	12	1.43	–
Binomial	P_*Mobula* spp._	year + SST anomalies* + offset (log (dive time))	2	–	0.8
	P_*T. meyeni*_	year* + SST anomalies* + offset (log (dive time))	34		0.96
	P_*S. lewini*_	year* + SST anomalies* + offset (log (dive time))	5	–	0.5
	P_*C. falciformis*_	year* + offset (log (dive time))	7	–	0.8
	P_*C. galapagensis*_	year* + season* + offset (log (dive time))	13	–	0.6
	P_*T. obesus*_	year* + SST anomalies* + offset (log (dive time))	10	–	0.9
	P_*C. limbatus*_	year + offset (log (dive time))	2	–	0.7
	P_*G. cuvier*_	year* + offset (log (dive time))	13	–	0.7

Asterisk indicates statistical significance, p < 0.05.

At 50–100 m, GAMs explained between 6 and 43% of the variation in the probability of occurrence for eight elasmobranch species that were sighted in at least 5% of the shallow dives (Table 2). The selected models demonstrated reasonably accurate predictions of the probability of occurrence for all species except *S. lewini*. While the statistical significance of the explanatory variables varied between species, the number of days since the start of the study was found to be significant (p < 0.05) for all species (Table 2). Similarly, binomial GLMs explained between 3 and 34% of the variation in the probability of occurrence of elasmobranch species (Table 3). According to the GLMs, the temporal trend (year effect) was found to be significant (p < 0.05) for six of the eight species considered (Table 3), with the probability of occurrence decreasing over time for *C. falciformis* and increasing for *T. meyeni*, *S. lewini*, *C. galapagensis*, *T. obesus*, and *G. cuvier* (Fig. 4). *Mobula* spp. And *C. limbatus* also showed decreasing and increasing trends, respectively, but these were not statistically significant. The largest change in probability of occurrence was observed for *Taeniurops meyeni* and *G. cuvier*, with an increase from 0.8% in 2010 to 61% in 2019 and from 2% in 2010 to 28% in 2019, respectively (Fig. 4; Supplement S5).

The GAMs showed that warmer SST anomalies were associated with a significantly higher probability of seeing *S. lewini* and *T. obesus* during shallow dives (Table 2; Supplement S1). There was also a higher probability of occurrence of *Mobula* spp. during afternoon hours and a higher probability of occurrence of *C. galapagensis* during the wet season (Table 2; Supplement S1). In addition, the GLMs showed that the probability of occurrence of *T. meyeni*, *S. lewini,* and *T. obesus* increased significantly under warmer SST anomalies, whereas the probability of occurrence of *Mobula* spp. decreased (Table 3; Supplement S2).

**Figure 5:**
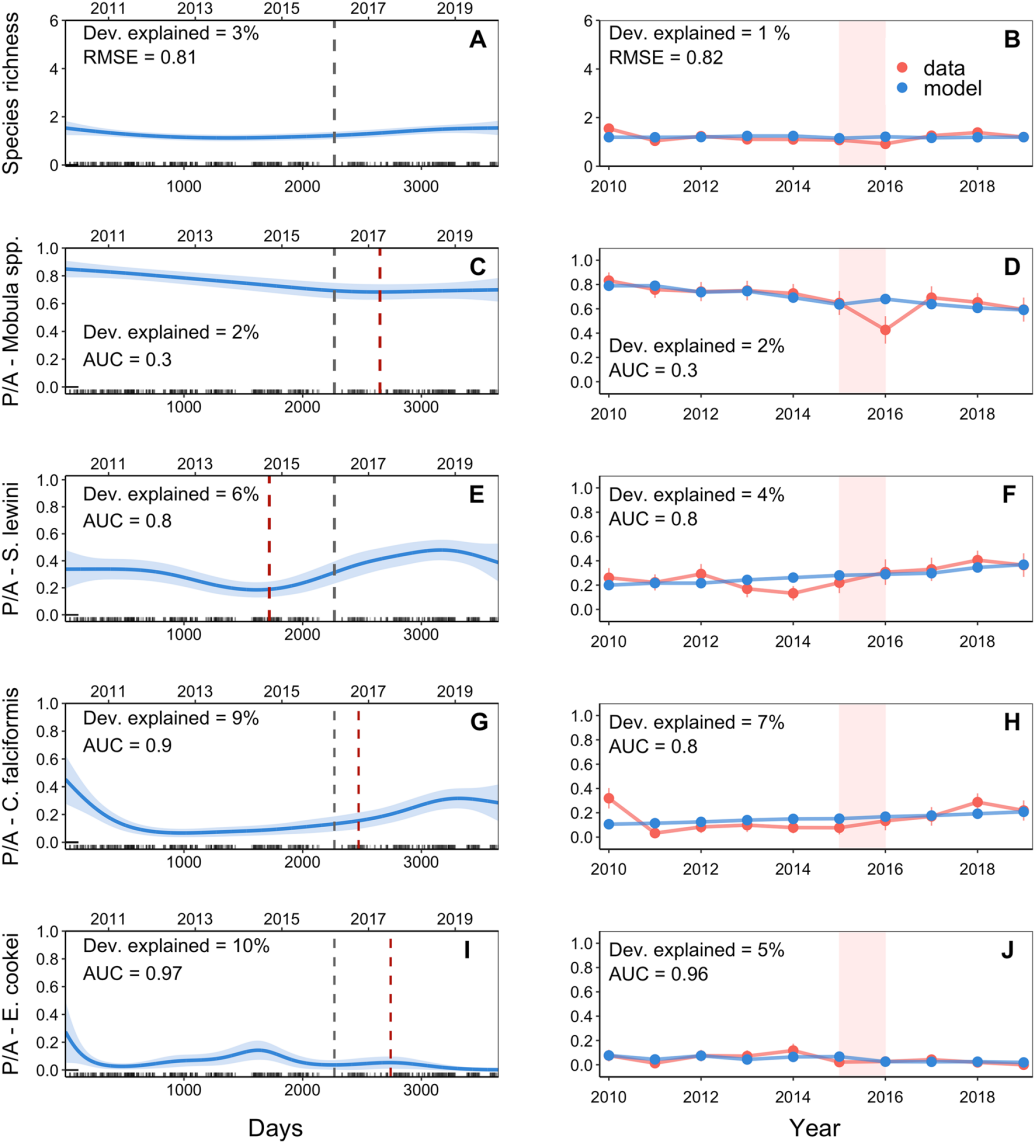
GAM estimates of daily species richness and probability of occurrence of elasmobranch species for depths between 300 and 400 m (**A**, **C**, **E**, **G**, **I**). The x-axis indicates the number of days relative to the start of the study (January 1, 2010). Estimated smooth functions (solid lines) with 95% confidence intervals (shaded areas) are shown for each numerical explanatory variable. The dates when a surveillance radar was installed on Cocos Island (dashed grey line) and when the species was listed in CITES Appendix II (dashed red line) are shown as reference points. Observed data and GLM model estimates of mean annual species richness and probability of occurrence of elasmobranch species from 2010 to 2019 for depths between 300 and 400 m (**B**, **D**, **F**, **H**, **J**). Bars indicate the 95% confidence intervals. Red rectangles indicate the El Niño event of 2015–2016.

For dives from 300 to 400 m, the GAM that provided the best explanation for species richness included the day and time period. However, it only accounted for 3% of the variation in the number of species observed per dive (Table 4). The predicted number of species remained relatively stable over time (Fig. 5A) and did not exhibit a significant linear trend across years (Fig. 5B). Only four elasmobranch species appeared in at least 5% of the deep dives, and GAMs explained between 2 and 10% of the variation in their probability of occurrence (Table 4). The GLMs explained between 2 and 5% of the variation in the probability of occurrence, with AUC values ranging from 0.3 to 0.96 (Table 5). The temporal trend (year effect) was found to be significant for the four species considered (Table 5), with the probability of occurrence decreasing for *Mobula* spp. and *E. cookei*, but increasing for *S. lewini*, and *C. falciformis.* For deep dives, *C. falciformis* and *S. lewini* showed the largest percentage change in probability of occurrence, increasing from 11% in 2010 to 21% in 2019 and from 20% in 2010 to 37% in 2019, respectively (Fig. 5; Supplement S5).

**Table 4:** Summary results Generalized Additive Models for species richness and presence-absence data of elasmobranch species sighted during deep dives (300–400 m) at Isla del Coco, Eastern Tropical Pacific Ocean.

Error distribution	Response	Predictors	% deviance explained	RMSE	AUC
Poisson	Richness	s(days)* + time period* + offset (log (dive time))	3	0.81	–
Binomial	P_*Mobula* spp._	s(days)* + time period * + offset (log (dive time))	2	–	0.3
	P_*S. lewini*_	s(days)* + SST anomalies* + time period* + offset (log (dive time))	6	–	0.8
	P_*C. falciformis*_	s(days)* + season* + offset (log (dive time))	8	–	0.7
	P_*E. cookei*_	s(days)* + time period* + offset (log (dive time))	10	–	0.97

Asterisk indicates statistical significance, p < 0.05.

**Table 5:** Summarized results of final selected binomial GLM models for species richness and presence absence data of elasmobranch species sighted during deep dives (300–400 m) at Isla del Coco, eastern Pacific Ocean.

Error distribution	Response	Predictors	% deviance explained	RMSE	AUC
Poisson	Richness	year + time period* + offset (log (dive time))	1	0.82	–
Binomial	P_*Mobula* spp._	year* + SST anomalies* + time period* + offset (log (dive time))	2	–	0.3
	P_*S. lewini*_	year* + season*time period* + offset (log (dive time))	4	–	0.8
	P_*C. falciformis*_	year* + offset (log (dive time))	1	–	0.8
	P_*E. cookei*_	year* + SST anomalies* + time period* + offset (log (dive time))	5	–	0.96

Asterisk indicates statistical significance, p < 0.05.

The probability of occurrence of *S. lewini* on deep dives increased with warmer SST anomalies (Table 4; Supplement S3). Warmer SST anomalies also had a positive effect on the probability of occurrence of *E. cookei*, and a negative effect on the probability of occurrence of *Mobula* spp. (Table 5; Supplement S4). There was a higher probability of occurrence of *Mobula* spp. and *S. lewini* during afternoon hours, while the probability of occurrence of *E*. *cookei* was higher in the morning. Additionally, there was a higher probability of occurrence of *C. falciformis* during the wet season (Table 4; Supplement S3).

### Drivers of depth distribution

**Figure 6:**
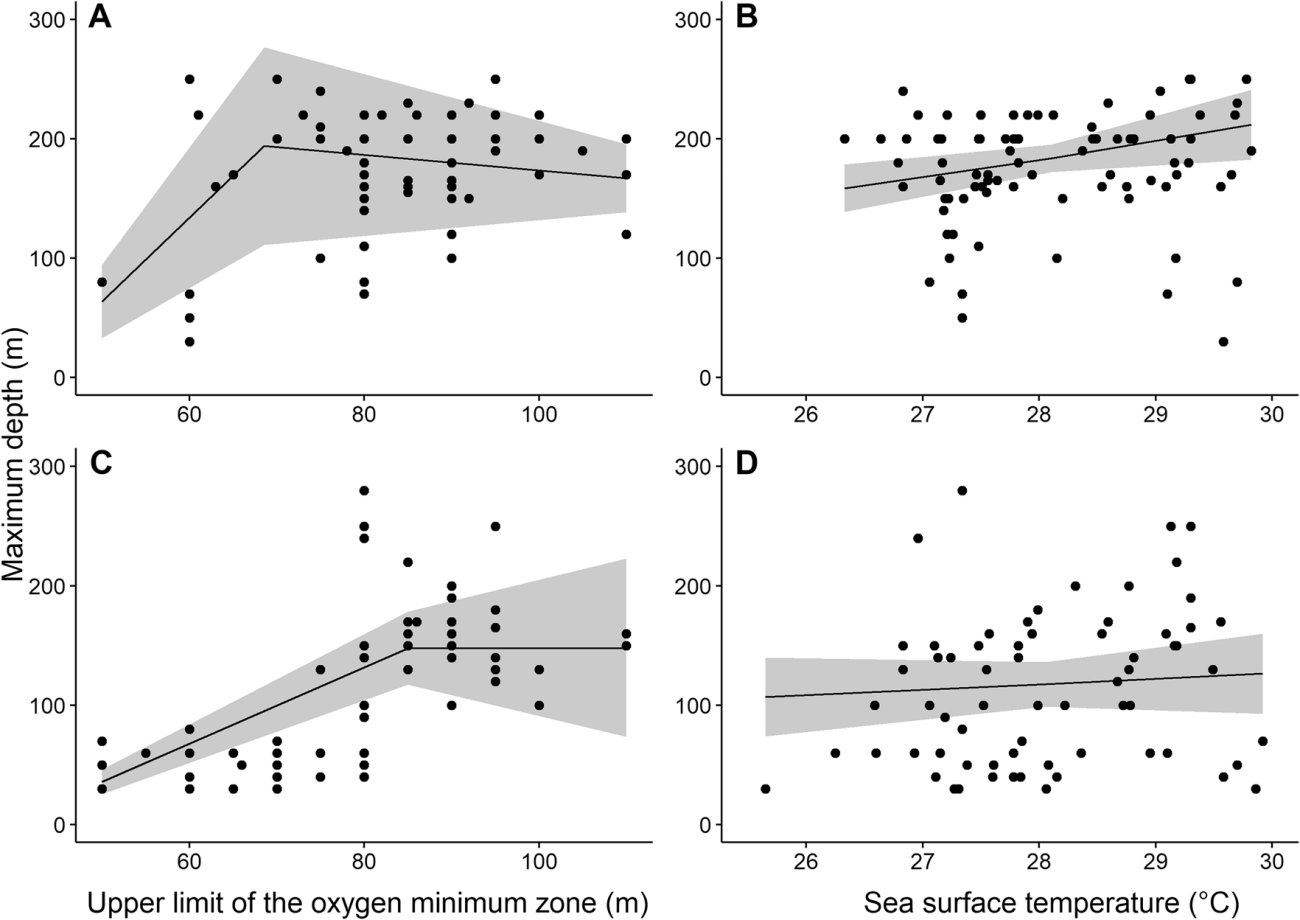
Relationship between the maximum depth of sightings and the upper limit of the oxygen minimum zone and sea surface temperature for *Mobula* spp. (**A**, **B**) and *Sphyrna lewini* (**C**, **D**).

Thermocline depth limits species depth ranges in waters shallower than 69 m (p = 0.002) for *Mobula* spp. and 85 m for *S. lewini* (p = 0.046) (Fig. 6). Both *Mobula* spp. and S.* lewini* were observed below the deeper limit of the thermocline, suggesting they can inhabit the upper boundaries of the OMZ. There was a positive relationship between SST and species maximum depth, indicating that warmer waters at the surface may force species to shift towards deeper waters. However, this correlation was only statistically significant for *Mobula* spp. (p = 0.018) (Fig. 6). Sea surface temperature and the deeper limit of the thermocline explained 52% in the variance of the maximum sighting depths for *S. lewini*, and 20% of the variance in the maximum sighting depths for *Mobula* spp.

## Discussion

This study shows that well-designed, long-term citizen science efforts can serve as a baseline to monitor elasmobranch species in deepwater habitats from data-poor regions where scientific surveys are often limited. Our findings revealed that from 2010 to 2019, some elasmobranchs have experienced significant declines in their probability of occurrence, while others exhibited large interannual fluctuations or increases over time. Furthermore, our results revealed that environmental drivers, such as sea surface temperature/warming, and the upper boundary of the oxygen minimum zone influence habitat preferences and occurrence patterns of elasmobranchs in Isla del Coco. This study underscores the importance of long-term data sets to understand species population trends and shed light on the potential impacts of human activities and the rising impacts of climate change on elasmobranch populations.

A decade of observations from the *DeepSee* submersible found a total of 17 elasmobranch species at Isla del Coco, including deepwater species such as the Smalltooth sand tiger shark (*Odontaspis ferox*), the Prickly shark (*E. cookei*), and the Chilean torpedo (*T. tremens*), which would have remained inaccessible without the use of a submersible or ROV^23,25^. Most elasmobranch surveys on Isla del Coco have been limited to relatively shallow (< 30 m) coastal habitats, providing a limited view of species composition and population trends over a relatively small depth range^9,19^. For example, recent surveys using baited remoted underwater video stations (BRUVS) and diving observations in shallow habitats around Isla del Coco reported 12 and 14 elasmobranch species, respectively^9,21^. Our study also found a higher elasmobranch species richness in shallow (50–100 m; n = 361) than deep waters (300–400 m; n = 1141). Elasmobranchs were also more likely to be seen on shallow than deep dives. The shallow waters around tropical oceanic islands such as Isla del Coco are highly diverse and productive, supporting a substantial biomass of large predatory fish^26,27^, while deep waters generally have fewer elasmobranch species and lower biomass due to challenging conditions (e.g., the presence of an oxygen minimum zone, low temperature, food availability, etc.)^28^. Species may be using these depths during dives where they “hold their breath”, and therefore, use these waters temporarily to thermoregulate or find prey, independent of low oxygen levels^29^.

Based on our findings, filter-feeder rays (*Mobula* spp.) were relatively more common in deeper waters, which is consistent with other studies from the Pacific and Indian Ocean^30,31^. *Mobula japonica* and *M. tarapacana*, for example, are known to exhibit oscillatory vertical movements, diving deeper (> 100 m) more frequently during the day and rising to the surface at night, most likely in search of plankton concentrations to feed on^31,32^. The vertical migration of zooplankton occurs with biomass movement towards the surface in the evening and towards deeper waters at dawn, and as has been suggested, most of *M. birostris* dietary intake is from mesopelagic origin^33^. An increase in the intensity and frequency of changes in environmental conditions could affect the daily and seasonal distribution of plankton, and as a result distribution and foraging dynamics of mobulid rays^34^. Other species like *S. lewini* and *C. galapagensis* were mainly recorded in rocky reef habitats at depths less than 100 m, most likely because they tend to aggregate in shallow habitats near oceanic islands or at seamounts during their migratory routes^35,36^. These rocky reefs, that emerge from large soft bottom areas, accumulate most of the biomass in the mesophotic zone of Isla del Coco and sharks are commonly observed hunting at the top of the pinnacles^24^. In addition, *S. lewini* showed increased sensitivity to surface warming and low oxygen levels shallower than the upper boundary of the oxygen minimum zone (100–150 m), which suggest conditions around 100 m depth may be favorable for the species^29,37^.

We recognized that underwater visual survey methods (e.g. diving surveys, BRUVS, submersibles, etc.) have their own set of limitations, as they typically represent a snapshot in time/space and can underestimate the true presence and abundance of a species (e.g. species moving outside the visual range of the observer or underwater camera, visibility conditions affecting the detectability of marine species, etc.)^9,38^. It is also unknown to what extent the use of mobile survey approaches such as the *DeepSee* submersible, which emit noise and are equipped with artificial lights, may alter the behavior of mobile marine organisms, either as an attractant or as an avoidance stimulus^39^. Furthermore, although there were some records of species abundance available, most of *DeepSee*’s dives were recreational and pilots were only required to record species occurrences. While there are limitations with species occurrence data, this metric does provide useful information of species distribution over time that we can relate to other factors. Long-term deepwater surveys such as the one presented in this study provide an important baseline to monitor changes in species occurrence over time. The frequency of sampling events from the *DeepSee* submersible (within and across years) in the same dive sites, covering different depths and periods of the day, offers a unique opportunity to examine population-level changes of elasmobranchs in response to common threats such as overfishing, habitat degradation and climate change.

### Temporal trends and changes in depth distributions

**Figure 7:**
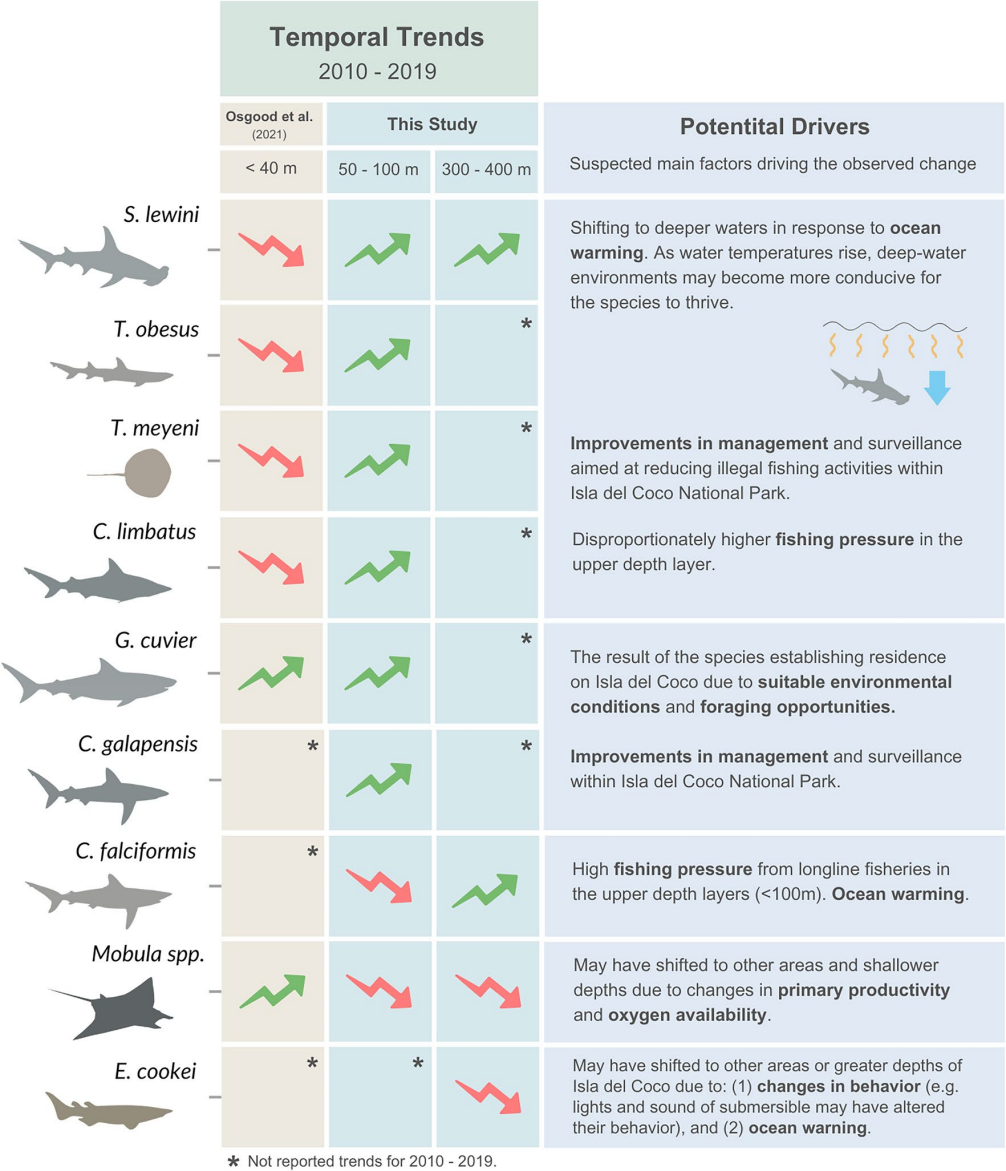
Temporal trends and potential drivers of the observed changes in common elasmobranch species recorded by the *DeepSee* submersible and previously reported by Osgood et al.^19^ for surface waters (< 40 m) of Isla del Coco (2010–2019).

Between 2010 and 2019, we observed a significant increase in the probability of occurrence of *T. meyeni*, *C. galapagensis*, *T. obesus, S. lewini* and *G. cuvier* in shallow dives (50–100 m), and of *S. lewini* and *C. falciformis* in deep dives (300–400 m) (Fig. 7). These increasing trends may be due to improvements in management and surveillance aimed at reducing illegal fishing activities within Isla del Coco National Park^40^. For instance, the introduction of a radar system in April 2016, capable of detecting vessels within the MPA’s 12-mile boundary, has likely increased the conservation benefits for elasmobranchs closely associated with Isla del Coco, including *T. meyeni*, *C. galapagensis* and *T. obesus*. In addition, over the past decade, Costa Rica has adopted other important regulations (e.g., several elasmobranch species have been listed in CMS and CITES appendices) that may be important for the recovery of elasmobranch populations, particularly highly mobile and migratory species that often receive limited protection from small and isolated MPAs, such as *S. lewini*^41,42^.

The increasing trends we found for some elasmobranch species contrast with previously reported trends in their abundance and occurrence in surface waters (< 40 m) of Isla del Coco^19^ (Fig. 7). Osgood et al.^19^ found that the abundance or probability of occurrence of *S. lewini, T. obesus*, *T. meyeni, and C. limbatus* declined in surface waters (< 40 m) between 2010 and 2019, yet our data indicate that the probability of occurrence of the same species increased in shallow dives (50–100 m). These contrasting patterns suggest that species may be shifting their distribution towards deeper waters. In addition, comparisons of the magnitude of change in the probability of occurrence or abundance of elasmobranch species between 2010 and 2019 at depths < 40 m (based on Osgood et al.^19^), 50–100 m (this study), and 300–400 m (this study) indicate that most species experienced a higher probability of occurrence towards deeper waters (Supplement S6).

Warming may be one of the factors driving elasmobranch species to shift their distribution towards deeper waters. For instance, Osgood et al.^19^ observed fewer *T. meyeni, T. obesus,* and *S. lewini* in surface waters (< 40 m) during strong El Niño events (2015/2016) when SST increased considerably, whereas in our study the occurrence of these species increased. Additionally, SST warming events were associated with a higher species richness recorded per dive at depths between 50 and 100 m. These findings imply that as water temperature rises, the environment is becoming more conducive for some species to thrive. This is because the local temperatures are getting closer to optimal levels, making the conditions more favorable, and thus increasing the chances of species occurrence^43,44^. In the case of reef-associated rays like *T. meyeni*, moving to deeper waters may allow them to remain within their optimal thermal niche. Other studies have reported that some rays have narrow thermal niches and limited ability to acclimatize^45^. *Triaenodon obesus* can tolerate high temperatures^19^, but as shallow waters become warmer, the species may move deeper to occupy more suitable habitats.

Warming-induced shifts in the depth distribution of *S. lewini* may explain the increasing trends we recorded for this species, which contrast with the previously reported decline in abundance in shallow waters of Isla del Coco^19,21^ and other oceanic islands in the Eastern Tropical Pacific Ocean^46^. Our results suggest that the depth distribution of *S. lewini* is being compressed, rather than shifting towards deeper waters. While warming is making surface waters less suitable, the upper boundary of the oxygen minimum zone may be acting as the deeper limit for this species’ distribution. *Sphyrna lewini* appears to be highly sensitive to the low oxygen waters within the oxygen minimum zone and prefers oxygenated waters above it. The shoaling oxygen minimum zone may be causing habitat losses at the deeper end of *S. lewini*’s distribution, as has been shown for billfish in the Eastern Tropical Pacific Ocean^47^ and for blue sharks in the oxygen minimum zone of the Atlantic^48^.

Our study revealed a significant increase in the probability of occurrence of *G. cuvier* over time, which is consistent with previous studies that have found similar trends for this species in the shallow waters of Isla del Coco since the early 2000’s^19,21^. Before 2005, diving observations of *G. cuvier* in Isla del Coco were uncommon^21^. Although this species is known to be highly mobile and capable of long-distance migrations^49^, some individuals have been observed to exhibit year-round residence on isolated reefs^50^. An increase in the occurrence of *G. cuvier* in Isla del Coco does not necessarily reflect a broader population trend but is more likely the result of the species establishing residence on the island due to suitable environmental conditions and foraging opportunities^21^.

While fishing pressure remains high outside Isla del Coco, a species with relatively high intrinsic rate of increase and post-release survival like *G. cuvier* may have some advantages over other elasmobranch species^51^. In addition, based on recent shark landing data (2015–2021) for the Pacific of Costa Rica, *G. cuvier* does not appeared to be a common bycatch species, suggesting there is currently minimal interaction with pelagic fisheries^52^. There is also some evidence that *G. cuvier* may be pupping in or near Isla del Coco as both neonates and females with abdominal distensions indicative of pregnancy have been reported in the island^53^, which could further indicate an established residence of the species^49,50^.

*Carcharhinus galapensis* also appears to have established a residency on Isla del Coco in recent decades. Prior to 2000, Galapagos sharks were not observed on Isla de Coco^20^. However, between 2008 and 2013, their probability of occurrence in surface waters (< 40 m) increased significantly^20^. Our study revealed a significant increase in the probability of occurrence of *C. galapensis* in shallow dives (50–100 m) between 2010 and 2019, and, overall, it was the second most commonly observed shark species. These results contrast with those reported for other oceanic islands in the Eastern Tropical Pacific Ocean, where *C. galapensis* abundance has remained relatively stable since the early 2000s^54^. Suitable environmental conditions, foraging opportunities, and the protection provided by Isla del Coco National Park may be driving the increased occurrence of *C. galapensis* on Isla del Coco, especially considering that Galapagos sharks tend to exhibit a high degree of residency on oceanic islands^55^.

Despite the conservation benefits provided by the Isla del Coco National Park, our results indicate that the occurrence of some sharks and rays has decreased over the past decade. From 2010 to 2019, there was a decrease in the probability of occurrence of *C. falciformis* and *Mobula* spp*.* at depths between 50 and 100 m. Similarly, the probability of occurrence of *Mobula* spp. and *E. cookei* also decreased significantly at depths between 300 and 400 m during the same period. The declining trend observed for the Prickly shark (*E. cookei*) represents one of the first sources of temporal data for this species listed as Data Deficient by the IUCN Red List^56^. This shark is potentially vulnerable to localized depletion due to its high site fidelity and relatively small home range^57^; however, it is unlikely that the declining trends observed for *E. cookei* are attributed to fishing as they have only rarely been reported in fishery catches^56^. Previous reports in the ETP have listed only 11 individuals caught in the shrimp trawl fishery in Costa Rica^58^, three in the artisanal pelagic fishery in Ecuador^59^ and one in Mexico^60^. One possible explanation is that individuals have shifted their distribution to other areas of Isla del Coco where the *DeepSee* submersible is not currently operating. *Echinorhinus cookei* is known to have relatively small home ranges, but they exhibit strong diel patterns with both a horizontal and vertical component^57^. In the Monterey Canyon of California, for example, at night the species is more active and moves from deep waters (200–250 m) to shallow habitats (50 m) possibly for feeding^57^. As *E. cookei* can use deeper waters than the maximum rated depth of the *DeepSee* submersible^61^ we hypothesized that horizontal and vertical diel movements, as well as climate-related changes affecting Isla del Coco may have influenced temporal trends in the occurrence of this species. In addition, further studies should investigate whether the use of submersibles may alter the behavior of marine species known to aggregate such as *E. cookei*^57^.

Declines in the occurrence of *C. falciformis* are consistent with global and regional population trends reported for this species in the ETP^10,19,46^, which are mainly associated with increasing fishing pressure. *Carcharhinus falciformis* is the most landed shark in Costa Rica and the second most common species in the global shark fin trade^62,63^. According to Dapp et al.^63^, most of the catch in the Costa Rican longline fishery consists of juvenile *C. falciformis*, with adults making up less than 15% of the catch. Dapp et al.^63^ also found a significant decrease in the total length of individuals caught from 2004 to 2007 and from 2008 to 2010, indicating a decline in the spawning stock biomass.

Our results also reflect possible shifts in the depth distribution of *C. falciformis* towards deeper waters, as the occurrence of this species decreased at depths between 50 and 100 m but increased between 300 and 400 m. This could be attributed to high fishing pressure from the longline fishery, that typically operates in the upper depth (< 100 m) layers^64^. Changes in water temperature may also be a key factor driving these shifts, as the spatial distribution of pelagic sharks in the ETP is highly correlated with SST^64^. While we did not find significant relationships between SST anomalies and the probability of occurrence of *C. falciformis* in the depth levels examined, this species is capable of making dives of up to 550 m^65,66^.

Declines in the occurrence of *Mobula* spp. are consistent with local and global population trends reported for Manta and devil rays^10,67^. In the ETP, *Mobula* spp. are frequently caught as bycatch in purse-seine, longline, driftnet and gillnet fisheries^68^. These species are also susceptible to climate change as they prefer cooler and more productive waters, and have high levels of ecological specialization^69,70^. The presence of cranial retia in *Mobula* spp. suggests that they have high metabolic rates, which may increase their sensitivity to high temperatures^71^. Our study found that the occurrence of *Mobula* spp. decreased during warming events, likely reflecting distributional shifts in response to changing temperatures^19^. However, while the probability of occurrence of *Mobula* spp. declined at deeper water layers (50–100 m and 300–400 m), Osgood et al.^19^ reported a small increase in surface waters of Isla del Coco (> 40 m) between 2010 and 2019. In other words, the depth distribution of *Mobula* spp. is shifting in the opposite direction of climate shifts, which may be explained by a high sensitivity to expanding oxygen minimum zones, or by a dependence on surface plankton aggregations as a food source^72^.

Both *C*. *falciformis* and *Mobula* spp. are capable of long-distance migrations, highlighting the need for broader management measures beyond localized MPAs. For example, the establishment of marine corridors, international agreements, and fishing regulations in the high seas are crucial to reduce fishing efforts and rebuild populations of threatened elasmobranchs^73^. In the ETP, there are increasing efforts to expand the size of MPAs around oceanic islands and connect wide-ranging pelagic species by creating marine corridors.

Our research showed that citizen science efforts have the potential to improve our understanding of the population trends of elasmobranch species in deepwater habitats from remote oceanic islands, providing insights into how climate change may be affecting species distributions in the Eastern Tropical Pacific Ocean^9,19^. Our study suggests that ocean warming and the upper boundary of the oxygen minimum zone may compress the vertical distribution and occurrence of elasmobranch species in deepwater habitats of Isla del Coco. Some species like *S. lewini* may be shifting their distributions towards deeper waters in response to ocean warming but may also be sensitive to low oxygen levels at greater depths. These findings highlight the need for regional 3D environmental information and long-term deepwater surveys to understand the extent of shark and ray population declines in the ETP and other regions, as most data sets available have been limited to relatively shallow waters.

## Materials and methods

### Study site

Isla del Coco is a volcanic oceanic island located 550 km southwest from mainland Costa Rica and is the only sub-aerial summit of the Coco Submarine Volcanic Range^74,75^. Due to its high level of biodiversity and marine endemism, Isla del Coco was declared a National Park in 1978, but it was not until 2001 that all marine extractive activities were prohibited at 12 nautical miles around the island^75^. Coastal habitats around the island are characterized by a complex bottom morphology^76,77^, with soft and hard substrates, rich benthic habitats in deep waters and extensive coral reefs and coral communities at shallow depths^78^. The insular platform around the island presents a higher bathymetric variation in the southwest side with several islets and submerged pinnacles, whereas the northeast side has a shorter insular platform with a higher slope^77,79^. Islets and rocky outcrops surrounding the island promote highly productive habitats by increasing vertical nutrient-rich fluxes and trapping material^80^.

The dynamic environmental variability in Isla del Coco is attributed to the southern oscillation of the Intertropical Convergence Zone, which determines the degree of influence of the North Equatorial Countercurrent, the main west–east current near to the equator^81^. There are two well-defined seasons, the wet (June–November) and dry (December–May) seasons^82^. During the wet season, the effect of the North Equatorial Countercurrent’s effect is heightened, leading to increased ocean productivity and currents, while also lowering water temperature^83^. Sea surface temperatures (SST) range from 24 and 29 °C, with temperatures known to increase during the El Niño Southern Oscillation (ENSO) events every 4–9 years^19^. As the result of high precipitation, salinity levels at Isla del Coco are among the lowest of all oceanic islands from the ETP, with the highest concentrations (33.27 psu) occurring during the dry season^82^. The upper boundary of the oxygen minimum zone (OMZ), where oxygen concentrations decline from 7 to 9 umol/kg, shoals up to 100–150 m beneath the surface^22^. The upper limit of this OMZ is dynamic, shoaling during strong upwelling phases like La Niña and deepening during weaker upwelling phases such as El Niño^22^. Climate change has led to an average increase in SST of ~ 1 °C in the ETP, which has coincided with the expansion of the oxygen minimum zone to shallower depths^22^.

### Frequency of elasmobranch occurrence

To examine general patterns in the frequency of occurrence of elasmobranchs at Isla del Coco, we compiled species presence/absence data logged from dives conducted onboard the *DeepSee* submersible between 2010 and 2019 (Fig. 1). The *DeepSee* is a custom built one-atmosphere submarine equipped with strong lights, a high-definition video camera and advanced navigation system, capable of carrying one pilot and two passengers to a maximum depth of 450 m and operational time of 6 h^84^. All elasmobranch species were recorded by submersible pilots that were trained on species identification from years of recreational diving observations and from the researchers leading scientific expeditions (Fig. 2). However, due to some initial concerns regarding the correct identification of manta and devil rays (*Mobula* spp.), these species were grouped together for further analyses. Given that depth records of some species were missing, we used the maximum depth recorded by the submersible during each dive as a proxy of the depth a species was sighted. Two depth intervals were used based on the most common maximum depths reached during the dives: (1) 50–100 m, and (2) between 300 and 400 m. Also, only dives that lasted for at least an hour were included since some of the dives that logged species were part of a training exercise for new submersible pilots. After filtering the database, a total of 1573 dives were included for analysis, of which 428 dives were considered shallow (50–100 m) and 1145 were considered deep (300–400 m).

### Temporal trends of elasmobranchs

We investigated temporal trends of common elasmobranchs at depths that are inaccessible to recreational SCUBA divers (> 50 m). Although the dataset had some abundance records, we used presence/absence data to avoid potential errors due to overestimation of elasmobranch abundance (recounting the same individuals observed during a dive) or variability attributed to different observers. In addition, the *DeepSee* submersible is mainly used for recreational dives with tourists, so most pilots typically record the species observed during their dives but not necessarily their abundance. Only during scientific expeditions or for a small proportion of recreation dives, pilots were logging abundance and species-specific depth records using standardized transects. We only considered recreational and scientific dives that were conducted in Everest, Piedra 165, and The Wall, as these sites were visited more frequently and had a higher quality of sighting records compared to other sites. These three sites were relatively close to each other; therefore, we did not consider any spatial variation or differences between sites, but rather focused on temporal trends of occurrence (presence/absence) at the two different depth levels (50–100 m, 300–400 m). After filtering the database, a total of 1502 dives were included for analysis, of which 361 were considered shallow (50–100 m) and 1141 were considered deep (300–400 m).

Generalized additive models (GAMs) were used to assess daily temporal changes in (1) species richness (i.e., number of elasmobranch species observed per dive), and (2) the probability of occurrence (i.e., presence/absence data) of individual species sighted at Isla del Coco between January 1, 2010 and December 31, 2019. Since the frequency of elasmobranch occurrence varied substantially between the two depth intervals considered, separate GAMs were constructed for shallow (50–100 m) and deeper (300–400 m) dives. Only species that appeared in more than 5% of the dives (shallow or deep) were included in the GAMs. We tested for the effect of days since the start of the study, time of day (morning: 8:00–12:00; afternoon: 13:00–16:00), season (dry: December to May; wet: June to November), and SST anomalies on (1) elasmobranch species richness, and (2) species occurrence (see Table 6). Of the total dives included for analysis, 763 occurred in the morning and 769 in the afternoon. Furthermore, 561 dives occurred during the dry season and 941 during the wet season (Supplement S7). Daily SST anomalies values were extracted from the NOAA’s Optimum Interpolation SST v2.1 (OISST v2.1)^85^. For elasmobranch species richness, we used GAMs with a Poisson distribution; for species occurrence, we used GAMs with a binomial distribution and logit link function. We applied smoothing parameters to the numerical explanatory variables that did not have a linear relationship with the response variables. The smooths were estimated using the restricted maximum likelihood method (REML). For all GAMs, we considered the duration of the dive as an offset variable to assess for variation in the elasmobranch species richness or occurrence due to changes in sampling effort. The models were developed using the gam function in the mgcv package in R^86^. We used the Akaike Information Criteria (AIC) to select the predictors that were included in the GAMs using the function dredge from the R package MuMIn^87^. Following^88^, the model evaluation was performed through 5 times cross validation based on training and test datasets created by a random selection of, respectively, 80% and 20% of the data. A residual analysis was also carried out for model validation (Supplements S8–S11). To assess the predictive performance of the species richness model (Poisson distribution), the root mean square error (RMSE) was calculated using the caret package in R^89^. For each species presence/absence model (Binomial distribution), we calculated the area under the receiver operating curve (AUC) using the MLmetrics package in R^90^. The AUC ranges from 0 to 1, with a value of 0.5 indicating as good as random performance, values between 0.7 and 0.9 considered useful, and values > 0.9 as excellent^91^.

**Table 6:** Environmental and temporal variables included in the GAM models.

Variable	Range	Description	Type of effect
Day	1–3651	The day of the dive relative to the start of the study, from January 1, 2010, to December 31, 2019	Fixed effect
Season	Two seasons (dry and wet)	Season of the dive	Fixed effect
Depth	Two depths categories (50–100 m or 300–400 m)	Classification according to the maximum deep reached in the dive [m]	Fixed effect
Anomalies	− 2 to 1.8 °C	Anomalies in SST (SST − average SST) [°C]	Fixed effect
Time period	Two time periods (morning and afternoon)	Time period of the day	Fixed effect
Dive time	60–250 min	Duration of the dive [min]	Offset

Generalized linear models (GLMs) were used to estimate linear temporal trends in (1) species richness and (2) the probability of occurrence of individual elasmobranch species over the study years. For the GLMs, the following predictors were included: year of the dive (2010–2019), time of day (morning: 8:00–12:00; afternoon: 13:00–16:00), season (dry: December to May; wet: June to November), and SST anomalies. For all models, the duration of the dive was considered as an offset variable. Like with the GAMs, separate GLMs were constructed for shallow (50–100 m) and deep (300–400 m) dives data. Model selection and validation followed the same steps described above for the GAMs.

### Drivers of depth distribution

We explored how the upper layer of the OMZ may be constraining the magnitude of depth shifts in elasmobranch at the Isla del Coco. The deep limit of the thermocline, used as a proxy for the upper boundary of the oxygen minimum zone was identified visually, and corroborated with temperature sensors onboard the submarine. The deep limit of the thermocline was documented in 283 dives where the maximum depth of *Mobula* spp. was recorded and in 253 dives where the maximum depth of *S. lewini* was recorded. We applied a segmented regression with a Gamma distribution to identify the depth range at which the OMZ constrains species distributions. This regression related the deeper limit of the thermocline (proxy for upper boundary of the OMZ) to the maximum depth at which the species was observed in each dive. The *segmented* R package was used for this analysis^92^.

### Ethics approval

### Supplementary Information

Supplementary Information 1.

Supplementary Information 2.

## Data Availability

All data generated or analyzed during this study are included as a Supplementary file [DeepSee dataset.csv].

## Code availability

The code written to organize and analyze data for this study is available from the corresponding author on reasonable request.
