# Supplementary material for: A decade of submersible observations revealed temporal trends in elasmobranchs in a remote island of the Eastern Tropical Pacific Ocean
Source: Sci Rep. 2024 Jun 14;14:13786. doi: 10.1038/s41598-024-64157-7 (PMC11178919; doi:10.1038/s41598-024-64157-7)
Supplement: Supplementary file 1 — Supplementary Information 1. [file 41598_2024_64157_MOESM1_ESM.docx]

A decade of submersible observations revealed temporal trends in elasmobranchs in a remote island of the Eastern Tropical Pacific Ocean

Mario Espinoza^1, 2, 3^, Fabio Quezada-Pérez^1^, Sergio Madrigal-Mora^4^, Beatriz Naranjo-Elizondo^1, 3, 5, 6^, Tayler M. Clarke^7^, Jorge Cortés^1, 2^

1 Centro de Investigación en Ciencias del Mar y Limnología, Universidad de Costa Rica, San Pedro, 11501-2060 San José, Costa Rica

2 Escuela de Biología, Universidad de Costa Rica, San Pedro, 11501-2060 San José, Costa Rica

3 MigraMar, Olema 94956, California, United States of America

4 California State University, Long Beach, California 90840, USA

5 Centro de Investigación en Estructuras Microscópicas (CIEMic), Universidad de Costa Rica, San Pedro, 11501-2060 San José, Costa Rica

6 Pelagos Okeanos, Moravia 11401, San José, Costa Rica

7 Changing Ocean Research Unit, Institute for the Oceans and Fisheries, University of British Columbia, AERL, 2202 Main Mall, Vancouver, BC Canada, V6T 1Z4

**Supplementary Material**


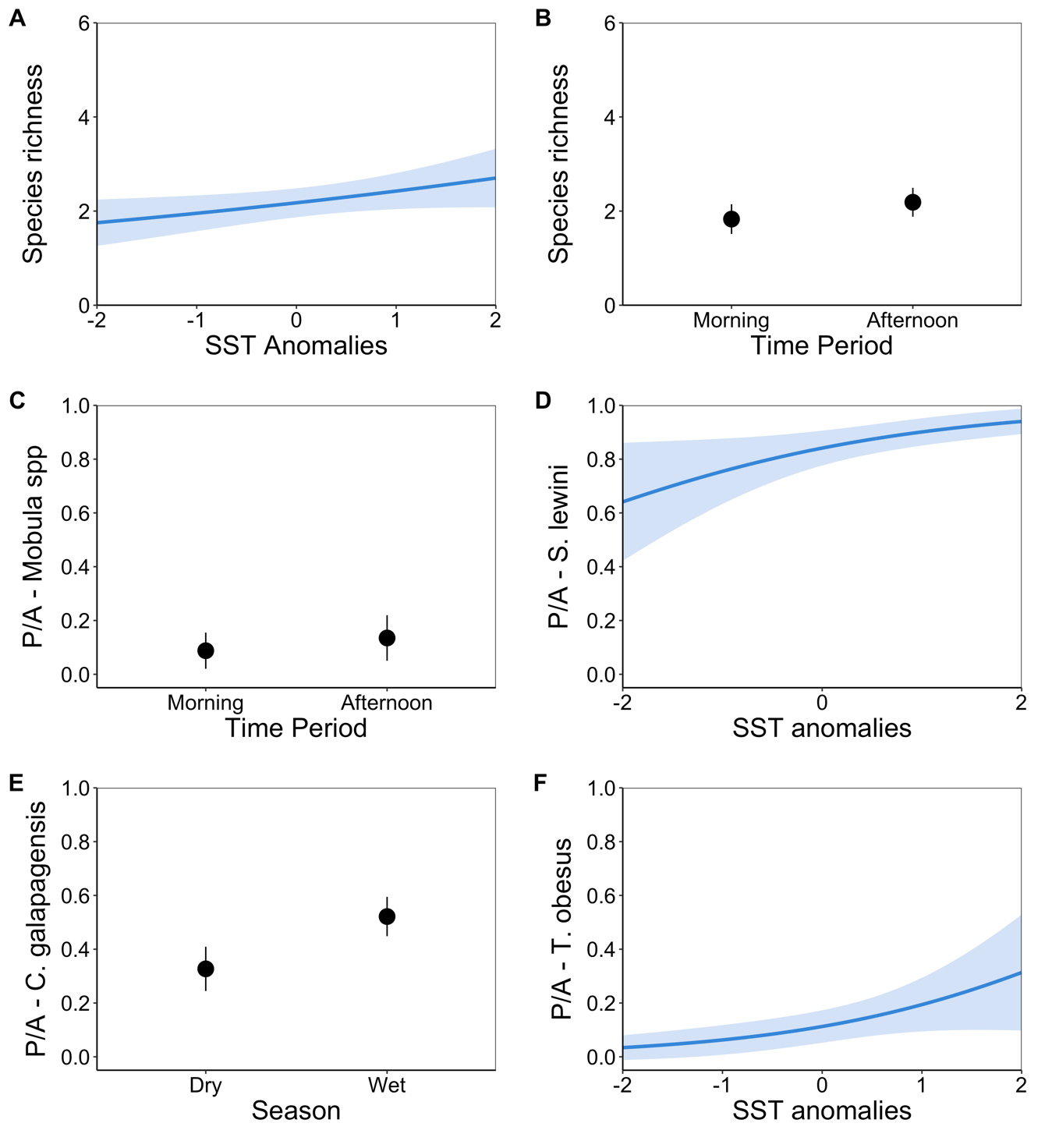


**Supplement S1.** GAM estimates of species richness and probability of occurrence of elasmobranch species sighted during shallow dives (50-100 m). Estimated smooth functions (solid lines) with 95% confidence intervals (shaded areas) are shown for each numerical explanatory variable. For the categorical variables, the bars represent the 95% confidence interval


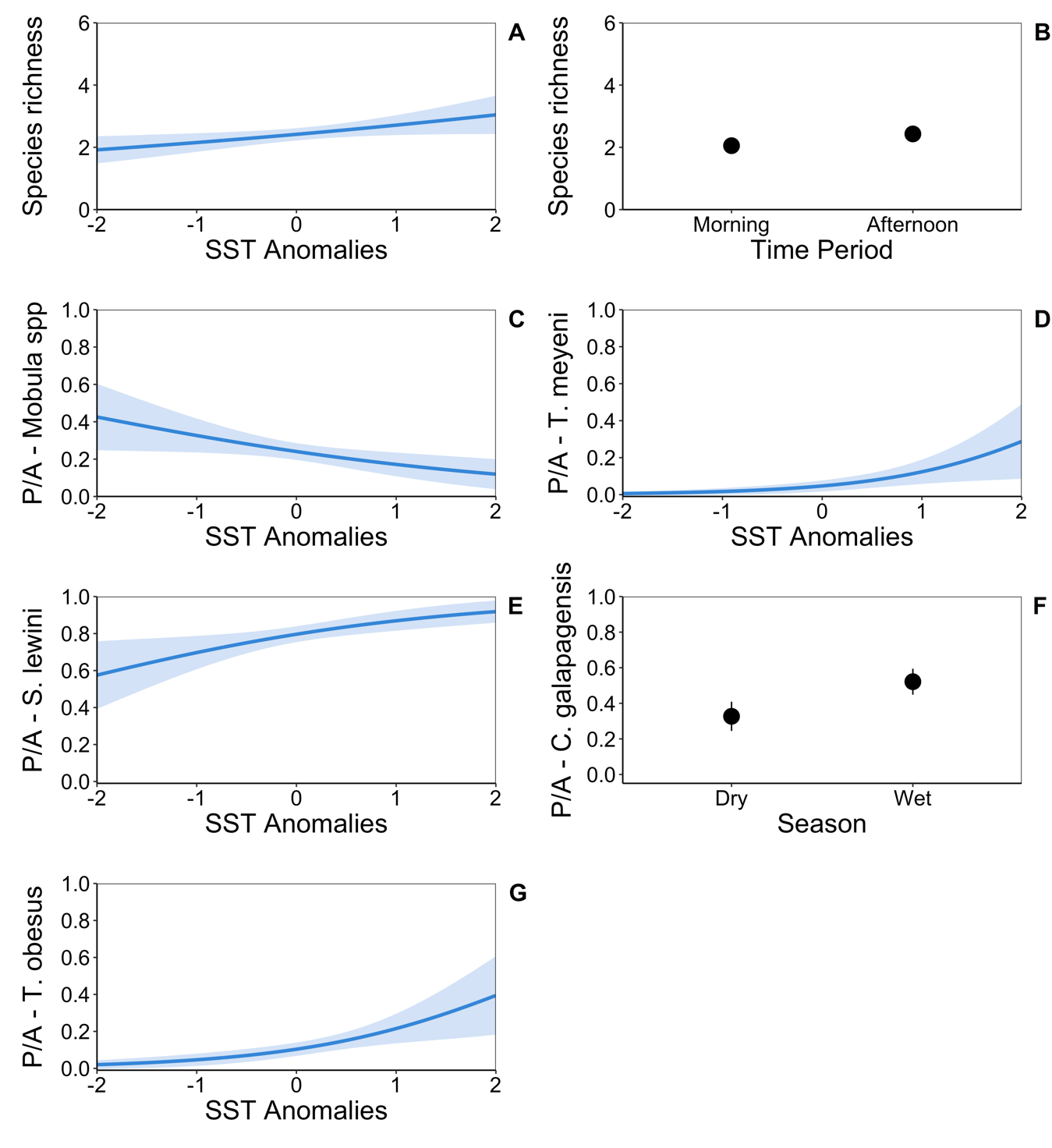


**Supplement S2.** GLM estimates of species richness and probability of occurrence of elasmobranch species sighted during shallow dives (50-100 m). Estimated smooth functions (solid lines) with 95% confidence intervals (shaded areas) are shown for each numerical explanatory variable. For the categorical variables, the bars represent the 95% confidence intervals.


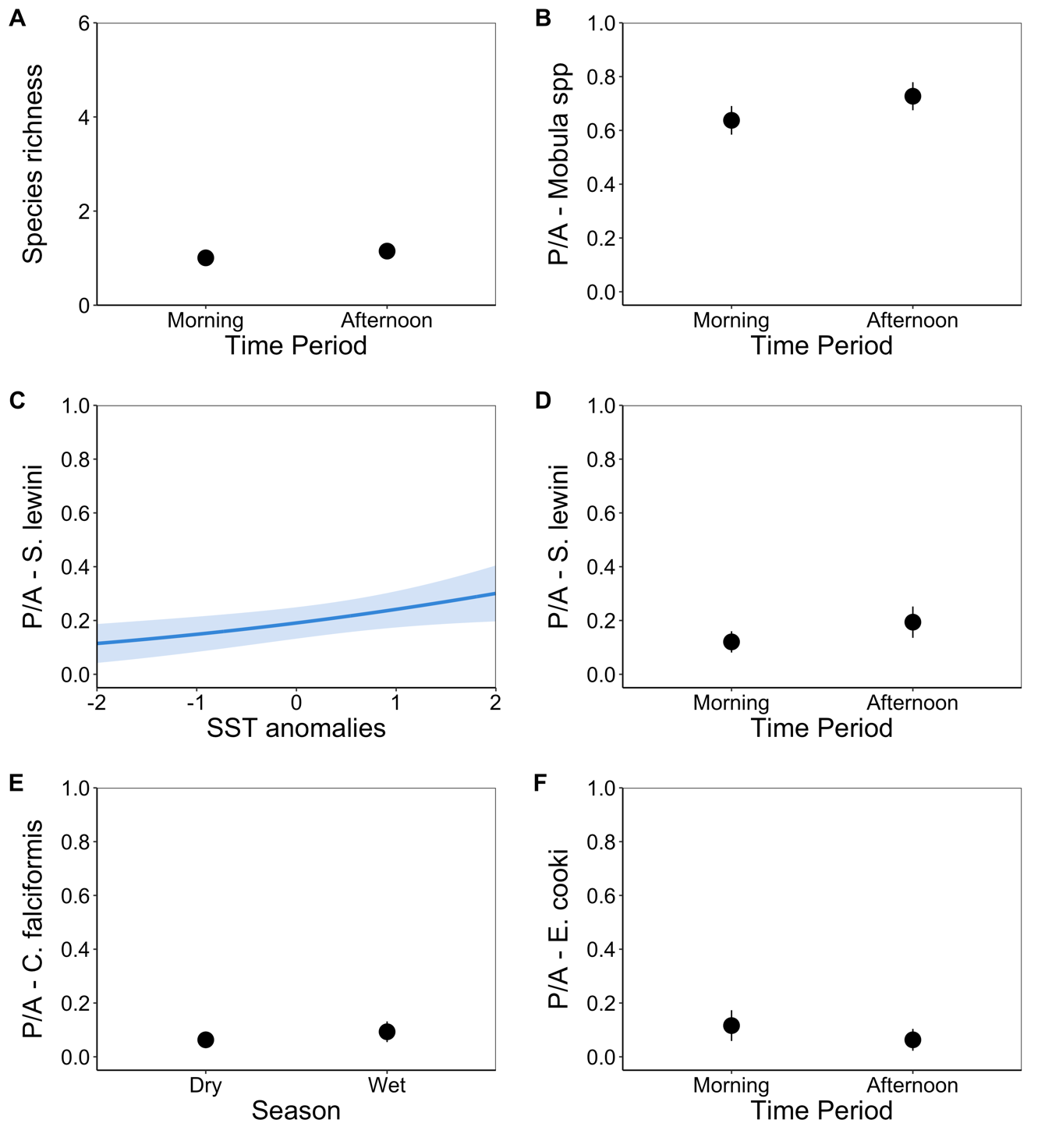


**Supplement S3.** GAM estimates of species richness and probability of occurrence of elasmobranch species sighted during deep dives (300-400 m). Estimated smooth functions (solid lines) with 95% confidence intervals (shaded areas) are shown for each numerical explanatory variable. For the categorical variables, the bars represent the 95% confidence intervals.


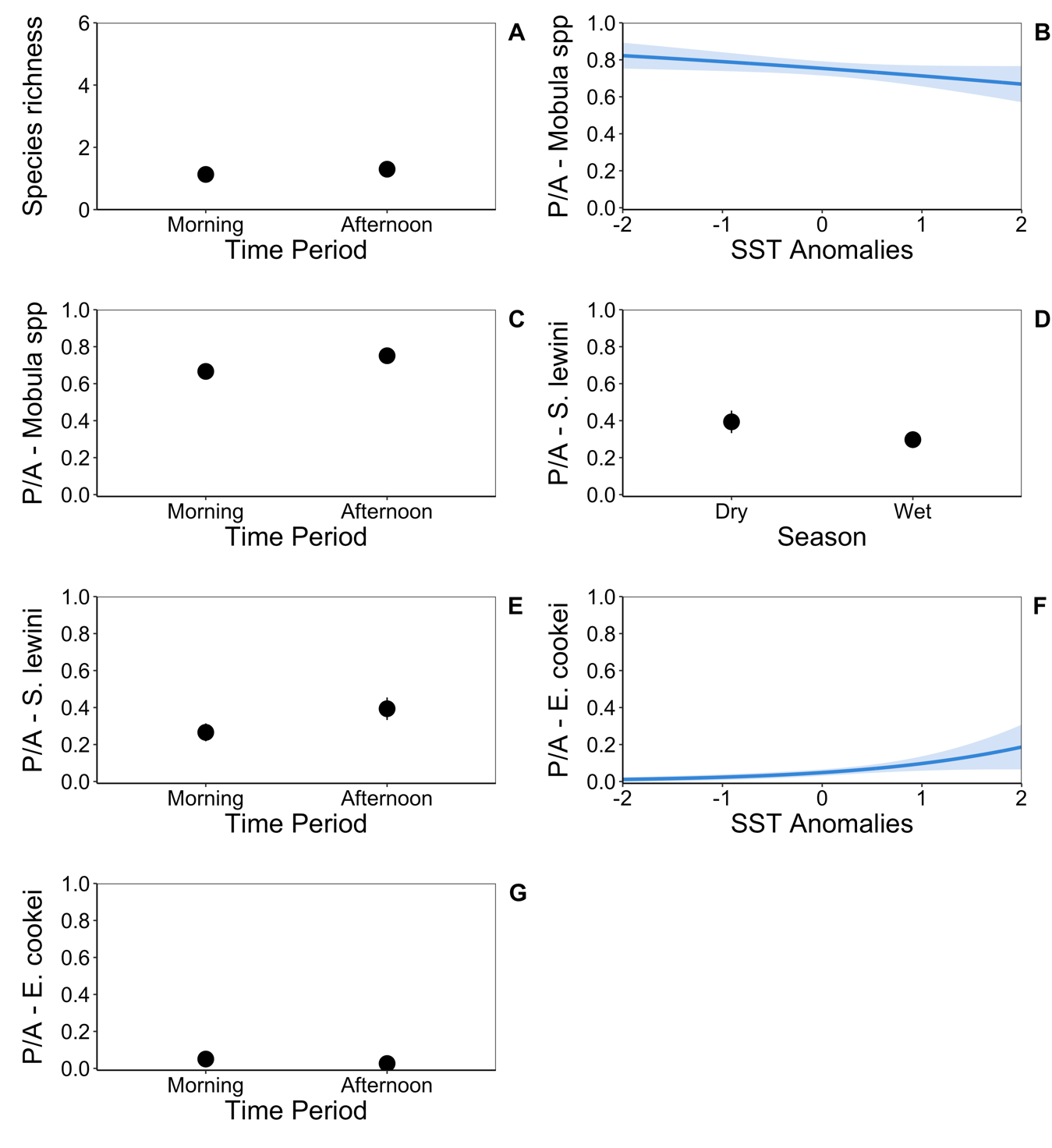


**Supplement S4.** GLM estimates of species richness and probability of occurrence of elasmobranch species sighted during deep dives (300-400 m). Estimated smooth functions (solid lines) with 95% confidence intervals (shaded areas) are shown for each numerical explanatory variable. For the categorical variables, the bars represent the 95% confidence interval.

**Supplement S5.** Percentage change in the annual mean probability of occurrence of elasmobranch species between 2010 and 2019 at Isla del Coco, eastern Tropical Pacific, for shallow (50-100m) and deep (300-400 m) dives, based on the binomial GLM models.

| Dive Maximum Depth | Species | Mean Prob. of Occurrence | | Percentage Change  % |
| --- | --- | --- | --- | --- |
|  |  | 2010 | 2019 |  |
| Shallow Dive | *T. meyeni* | 0.8 | 61 | 7525 |
| 50–100 m | *G. cuvier* | 2 | 28 | 1300 |
|  | *T. obesus* | 6 | 30 | 400 |
|  | *C. galapensis* | 23 | 78 | 239 |
|  | *C. limbatus* | 9 | 19 | 111 |
|  | *S. lewini* | 79 | 89 | 13 |
|  | *Mobula* spp. | 28 | 20 | -29 |
|  | *C. falciformis* | 49 | 10 | -80 |
| Deep Dive | *C. falciformis* | 11 | 21 | 91 |
| 300–400 m | *S. lewini* | 20 | 37 | 85 |
|  | *Mobula* spp. | 79 | 59 | -25 |
|  | *E. cookei* | 8 | 2 | -75 |
|  |  |  |  |  |


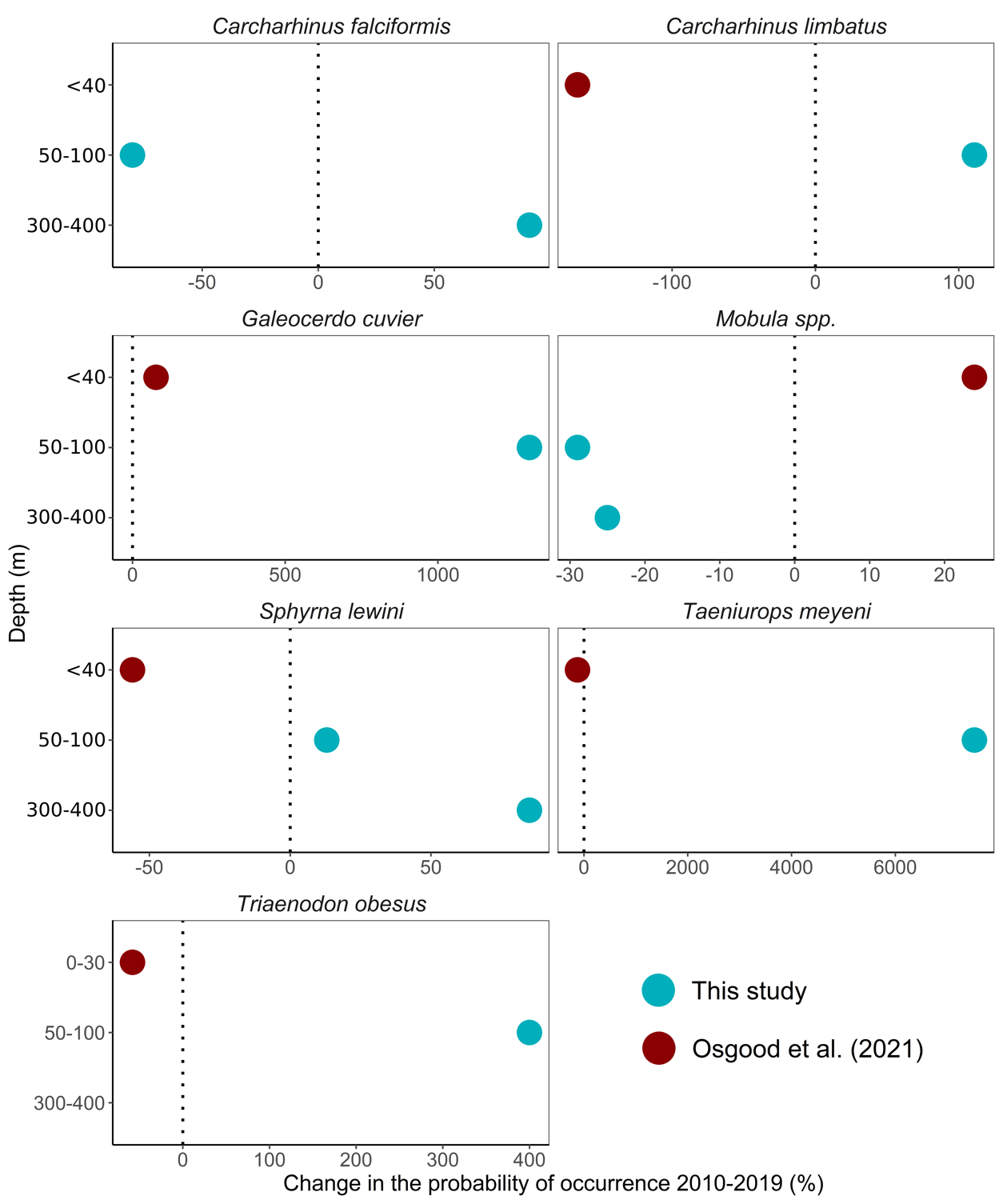


**Supplement S6**. Change in the probability of occurrence or change in abundance across depth levels for the seven most common elasmobranch species in the Isla del Coco. Change in the probability of occurrence at 50-100 m and 300-400 m was estimated in the present study for the 2010-2019 period. Change in the probability of occurrence or change in abundance at depths <40 m was estimated by Osgood et al. (2021) for the 2010-2019 period.


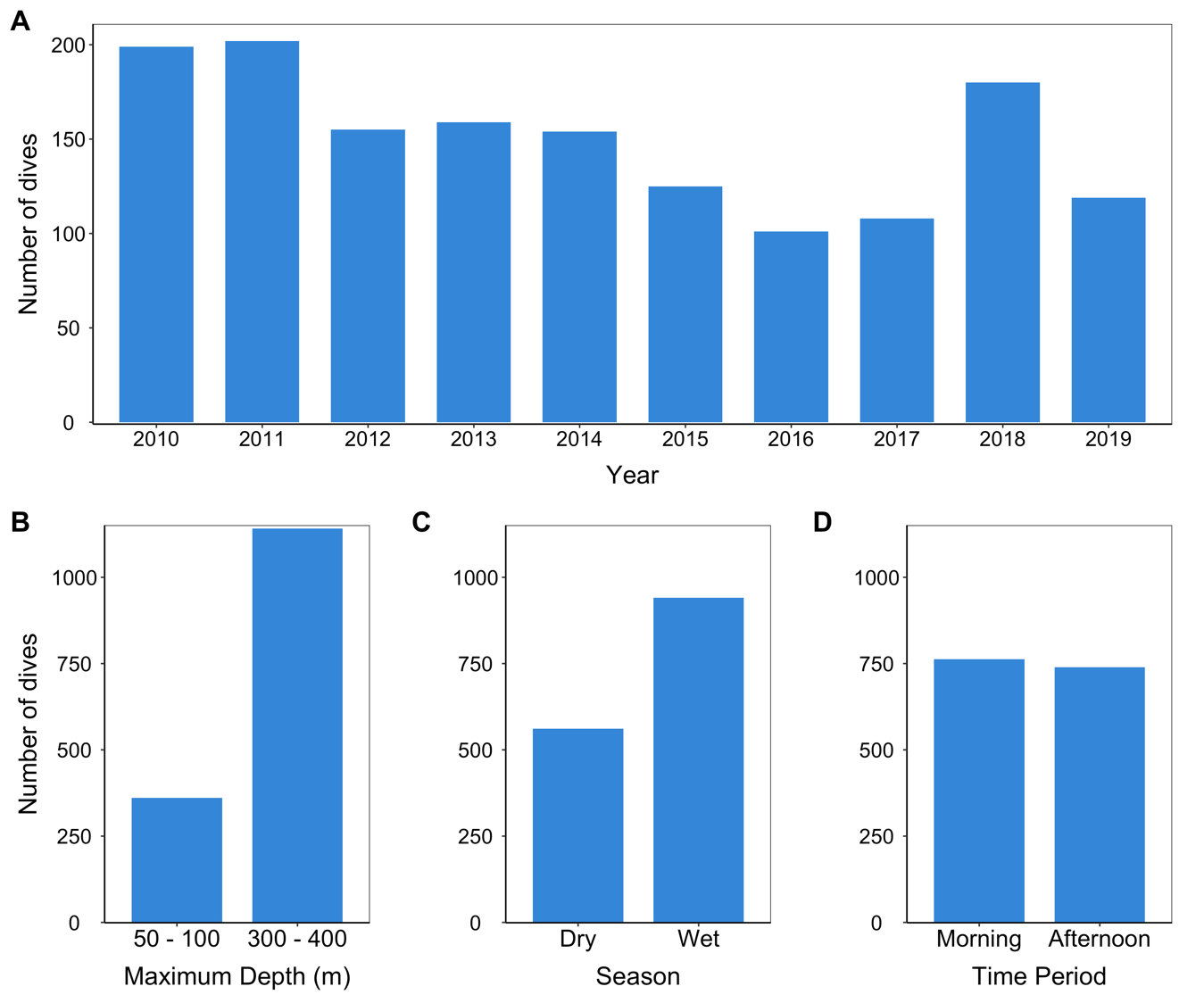


**Supplement S7.** Total number of *DeepSee* submersible dives at Isla del Coco considered in the GAMs and GLMs models by (A) year, (B) maximum depth range reached, (C) season, and (D) time period.

**
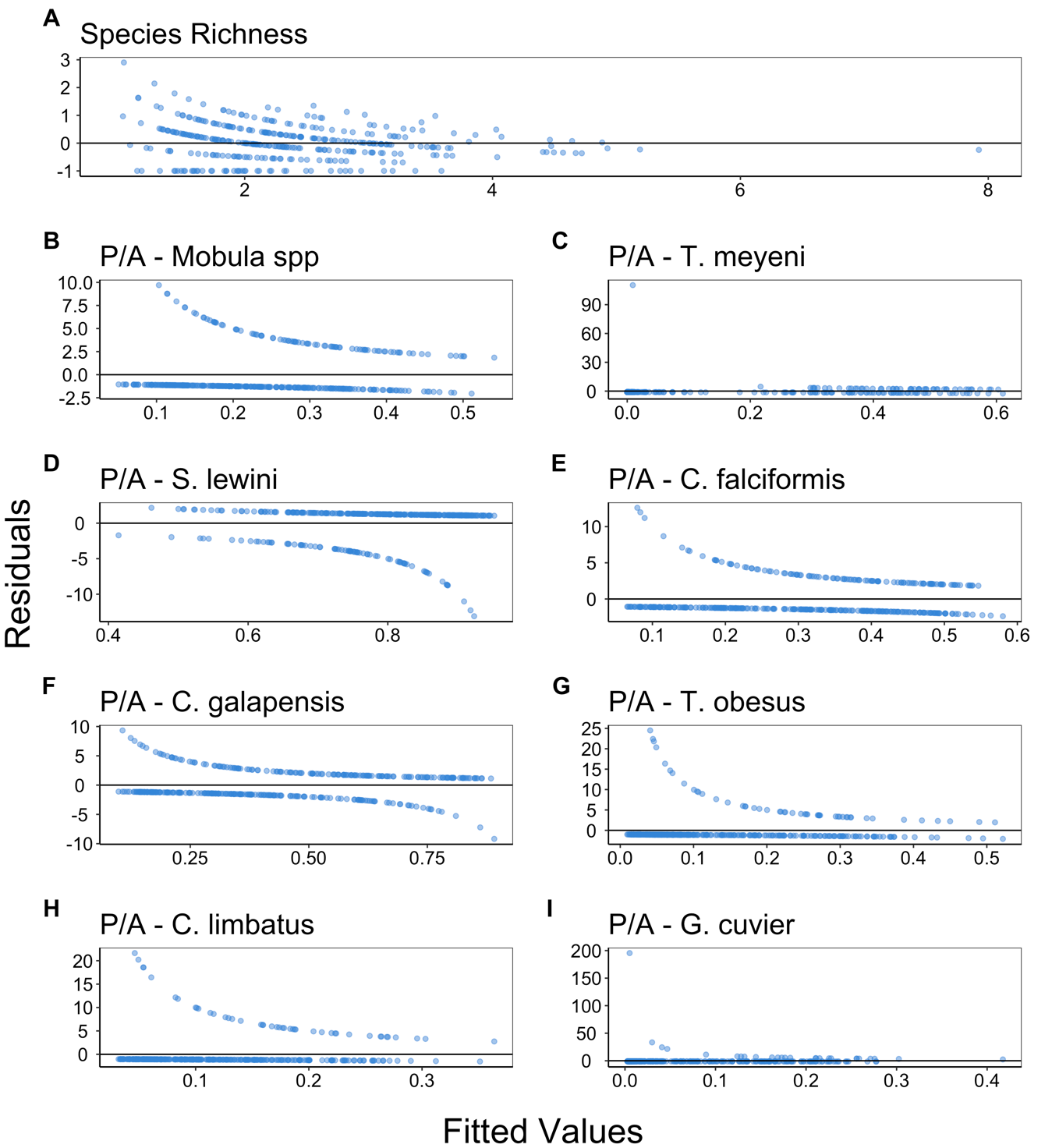
 Supplement S8.** Residual plots from GAMs of species richness and probability of occurrence of elasmobranch species sighted during shallow dives (50-100 m).


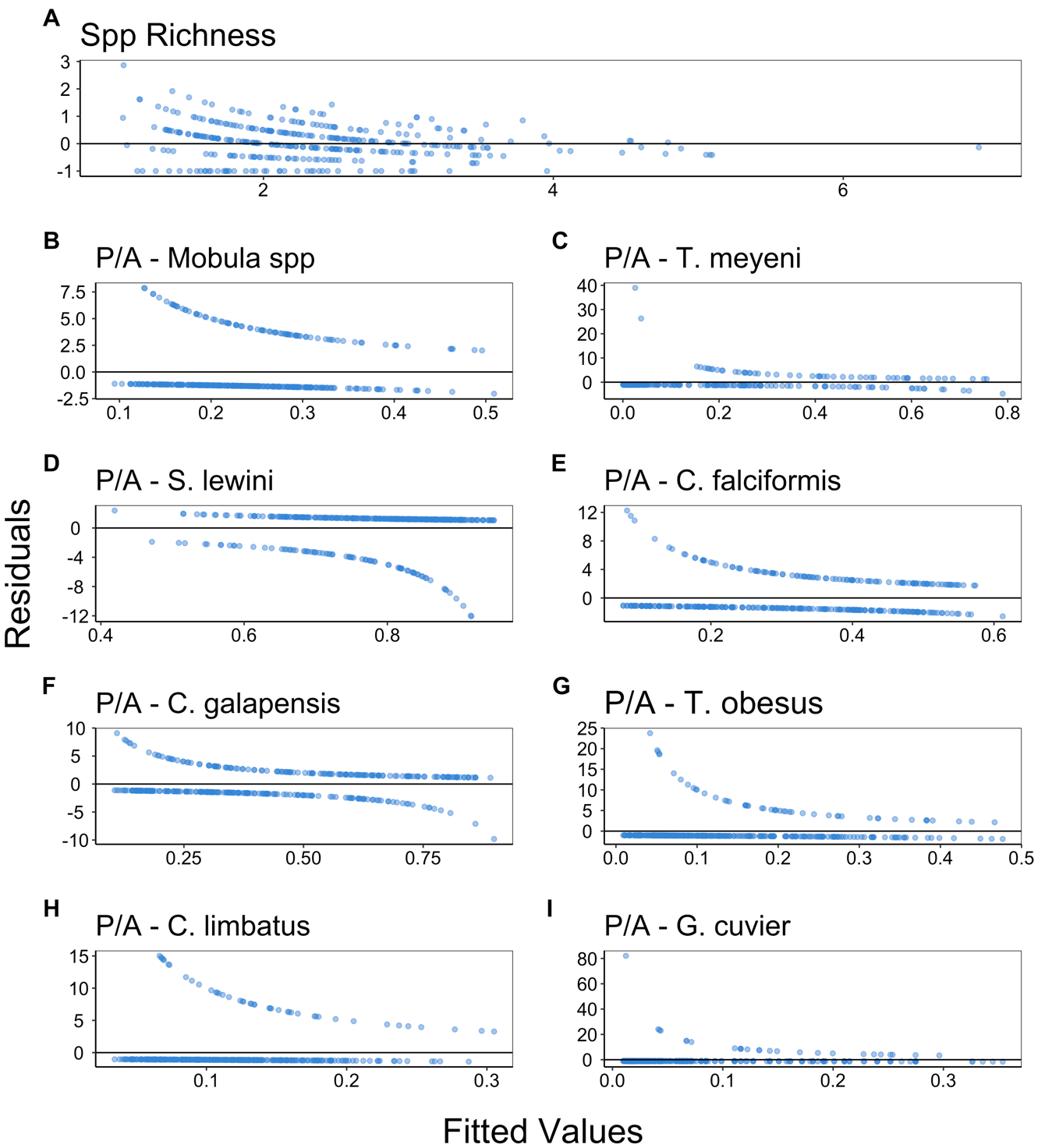


**Supplement S9.** Residual plots from GLMs of species richness and probability of occurrence of elasmobranch species sighted during shallow dives (50-100 m).


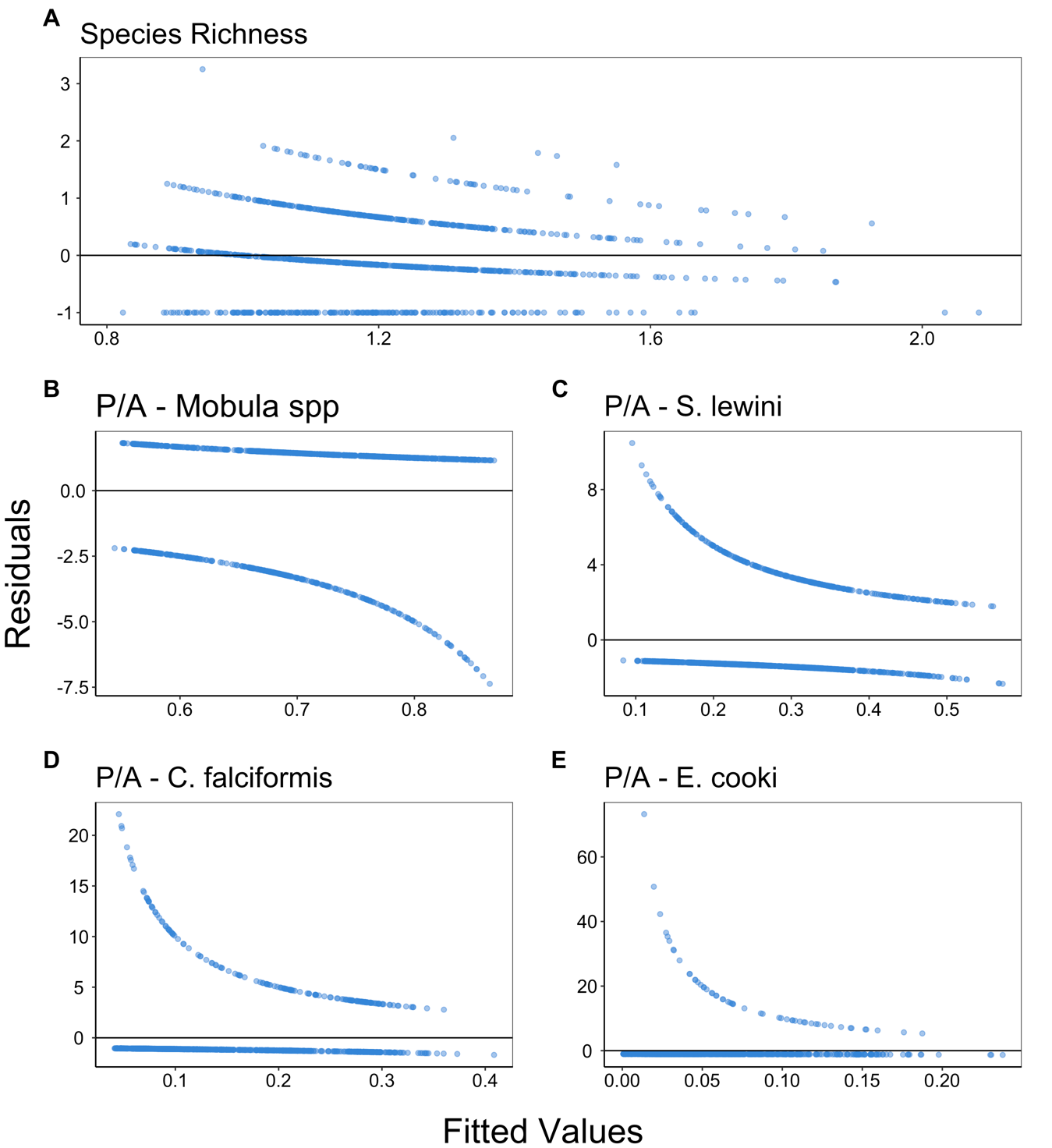


**Supplement S10.** Residual plots from GAMs of species richness and probability of occurrence of elasmobranch species sighted during deep dives (300-400 m).


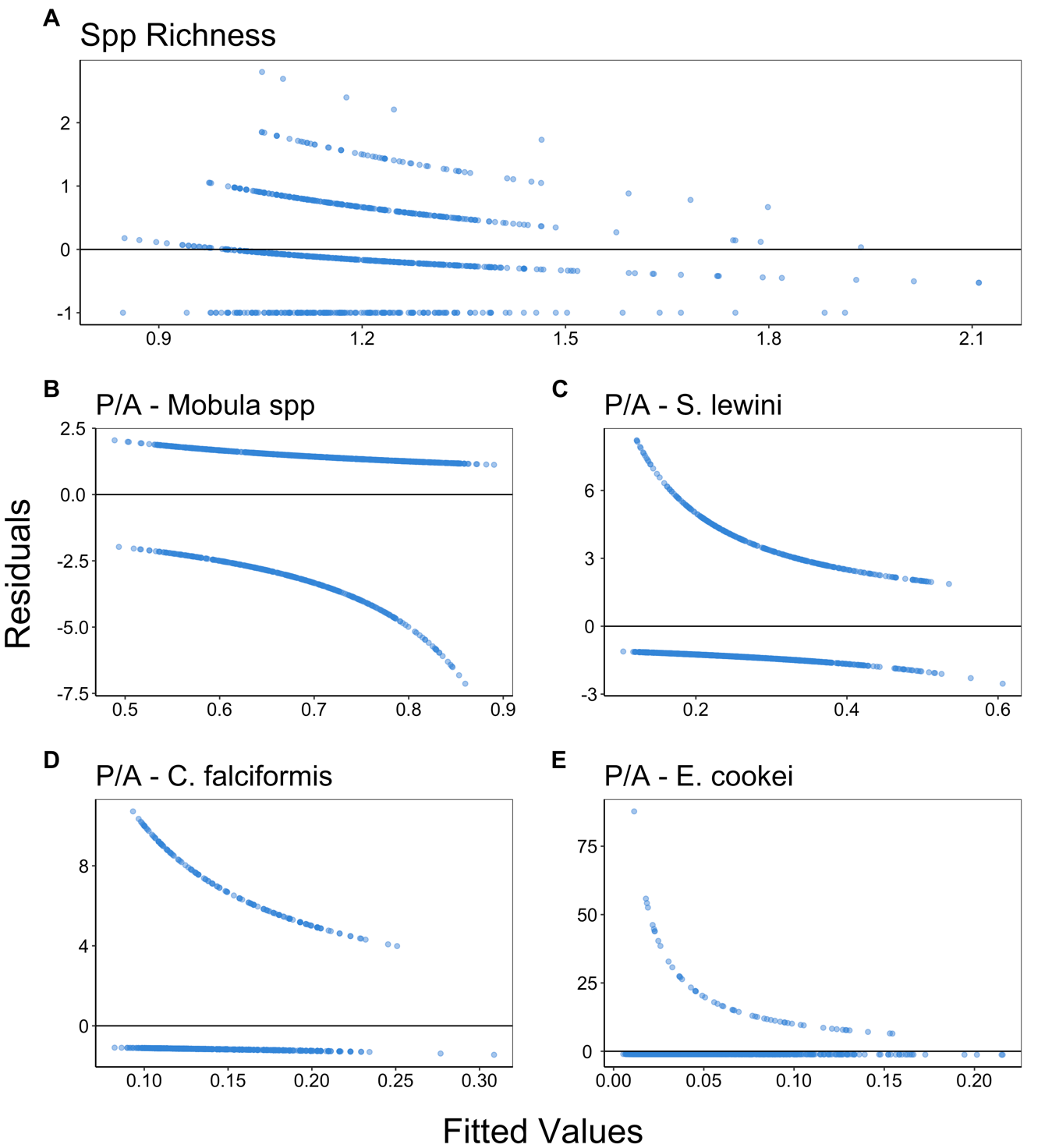


**Supplement S11.** Residual plots from GLMs of species richness and probability of occurrence of elasmobranch species sighted during deep dives (300-400 m).
